# The shifting landscape of asthma in the United States: An over 20-year analysis of prevalence, severity, and medication paradigms, 1999–2023^☆^

**DOI:** 10.1016/j.waojou.2026.101426

**Published:** 2026-07-02

**Authors:** Leiwen Fu, Ke Liu, Yuxia Du, Jian Du, Liang Li, Yang Liu, Weitao Su

**Affiliations:** a Beijing Chest Hospital, Capital Medical University, Beijing Tuberculosis and Thoracic Tumor Research Institute, Beijing, 101149, China; b Department of General Practice, The Second Affiliated Hospital of Fujian Medical University, Quanzhou, Fujian, 362000, China; c Central Laboratory, The Second Affiliated Hospital of Fujian Medical University, Quanzhou, Fujian, 362000, China

**Keywords:** Asthma, Prevalence, United States, NHANES

## Abstract

**Background:**

Asthma remains a substantial public health burden in the United States, warranting updated estimates of prevalence, morbidity, and medication use across population subgroups.

**Methods:**

This study analyzed data from 114,505 participants in the National Health and Nutrition Examination Survey (NHANES) from 1999 to 2023. Asthma status (lifetime, current, attack, emergency visit) and medication use were assessed through self-reports and prescription data. Weighted prevalence trends were evaluated across demographic subgroups.

**Results:**

Among 114,505 participants (median age 37 years; 51.2% male) in NHANES 1999–2023, the prevalence of lifetime and current asthma was 14.7% and 8.8%, respectively. Lifetime asthma prevalence increased from 11.2% to 15.8%, and current asthma from 6.5% to 9.3%, by 0.41% (95%CI: 0.28 to 0.54) and 0.24% (0.09–0.38) per NHANES cycle, respectively. Prevalence was consistently higher in women, non-Hispanic Black individuals, and those with lower socioeconomic status. Adolescents had the highest lifetime asthma prevalence (19.3%). Asthma attacks and emergency visits among those with current asthma declined significantly, by −0.99% (−1.94 to −0.03) and −1.94% (−2.96 to −0.93) per NHANES cycle, respectively. During the study period, the prevalence of asthma medication use showed a biphasic trend: an overall increase from 1999 to 2000 to a peak in 2007–2008, followed by a significant decline through 2020–2023. Lower education and income levels were associated with higher medication usage.

**Conclusion:**

Over the past 2 decades, asthma prevalence increased at the population level, whereas the prevalence of asthma attacks and asthma-related emergency visits among individuals with current asthma declined.

## Introduction

Asthma, a chronic inflammatory condition of the airways, remains a significant public health issue, with substantial implications for both individual health and healthcare systems.^1^^,^^2^ In the United States, asthma affects millions of individuals, contributing to considerable morbidity, healthcare expenditures, and lost productivity.^3^ According to recent report, approximately 25 million people in the United States had asthma in 2021, representing 7.7% of the total population.^4^ Of these individuals, 80% were adults and 20% were children. Despite significant advances in asthma treatment, it remains one of the costliest chronic conditions in the United States, imposing substantial economic burdens through direct medical costs, absenteeism, and premature mortality.^5^

The onset and exacerbation of asthma are influenced by a complex interplay of multiple factors.^6^ Environmental exposures, such as airborne allergens, air pollutants, and tobacco smoke, are positively associated with both the incidence and acute exacerbations of asthma.^7^ Respiratory viral infections, particularly early-life lower respiratory tract infections caused by respiratory syncytial virus or rhinovirus, may contribute to asthma development by damaging the airway epithelium, impairing antiviral immune responses, promoting type 2 airway inflammation, and facilitating airway remodeling. Previous studies have reported that children with a history of severe early-life respiratory syncytial virus lower respiratory tract infection have an approximately 2- to 12-fold higher risk of developing pediatric asthma.^8^^,^^9^ Family history is another important risk factor. Meta-analytic evidence suggests that maternal and paternal asthma are associated with approximately 3-fold and 2.4-fold higher odds of asthma in offspring, respectively.^10^

The pathogenic effects of these triggers exhibit significant demographic variability. Previous studies have shown that Puerto Ricans, Black, or African American populations are at a higher risk of developing asthma compared to other racial or ethnic groups.^11^ In childhood, asthma prevalence is notably higher in boys than in girls, however, the pattern reverses in adulthood, with women experiencing a higher prevalence.^12^ Moreover, older adults tend to exhibit more severe asthma symptoms and exacerbations. Economic factors may further exacerbate these disparities, with studies indicating that lower socioeconomic status is associated with higher asthma prevalence.^6^ Body mass index (BMI) and lifestyle factors may also play a role in influencing asthma onset.^12^^,^^13^

Previous National Health and Nutrition Examination Survey (NHANES)-based studies have provided important estimates of asthma prevalence and associated factors, particularly among adults.^14^ However, fewer studies have simultaneously assessed long-term national trends in lifetime asthma, current asthma, asthma attacks, asthma-related emergency visits, overall asthma medication use, and medication subclasses across all available age groups and major demographic and socioeconomic strata. To address this gap, the present study used NHANES 1999–2023 data to characterize temporal trends in asthma prevalence, severity-related indicators, and medication use in the United States population.

## Methods

### Study design and participants

The study leveraged data from the NHANES, an ongoing population-based surveillance program initiated in 1999 to monitor health and nutritional status across the United States.^15^ This stratified probability sampling framework incorporates geographic clustering and oversampling of minority subgroups to achieve nationally generalizable estimates aligned with Census demographic distributions. Full methodological documentation, including operational manuals and quality assurance procedures, is archived on the Centers for Disease Control and Prevention (CDC) website.^16^ The NHANES survey protocol was approved by the National Center for Health Statistics (NCHS) Ethics Review Board. Details regarding Institutional Review Board (IRB) approval for each survey cycle are available elsewhere.^17^ This study included participants with data on self-reported asthma from NHANES 1999–2023 (N = 114505).

### Demographic characteristics

Demographic data were obtained through structured questionnaires, including age (years), sex (male/female), race (Mexican American, other-Hispanic, non-Hispanic White, non-Hispanic Black, and other race), education level (less than high school, high school graduate, some college or AA degree, college graduate or above), and poverty-to-income ratio (PIR). To avoid age-related variability in educational attainment, education levels were analyzed only in adults aged ≥20 years. PIR, calculated as household income relative to federal poverty thresholds adjusted for household size and geographic location, was categorized into 3 groups: <1.3, 1.3–3.5, and >3.5. Individuals aged 16 and over responded to survey questions directly; for participants below the age of 16, or for those incapable of self-reporting, relevant information was supplied by a proxy respondent, who was typically a parent.

### Definition of asthma status

The medical conditions questionnaire recorded asthma status information. Participants were first asked: (1) “Ever been told you have asthma?” Those who answered “Yes” were subsequently asked: (2) “Still have asthma?” If affirmative, they were further questioned: (3) “Had asthma attack in past year” and (4) “Emergency care visit for asthma in past year?” Since the “Still have asthma” question in the 1999–2000 cycle was limited to participants under 20 years old, while asthma-related questions in other cycles had no age restrictions, current asthma, asthma attack in past year, and asthma-related emergency visits in past year were excluded from the 1999–2000 cycle to maintain consistency across cycles. Our analysis comprised prevalence of lifetime asthma (including both former and current asthma) and current asthma among all participants, as well as disease severity in the asthma population—specifically, prevalence of asthma attacks and asthma-related emergency visits in the past year among individuals with current asthma.

### Classification of asthma medications

During the interview phase, survey respondents were queried about their use of any prescribed medications within the preceding 30-day period. In instances of an affirmative response, interviewers documented the names of a maximum of 20 prescription drugs used within the previous 30 days, transcribing this information directly from the drug's packaging (eg, bottle, vial, inhaler, blister pack, paperboard box). When the medication's container was inaccessible, the interviewer noted the drug name based on the participant's recall. Subsequently, these medications were assigned to three-level therapeutic categories by employing the Multum Lexicon drug database, a system that offers a nested classification structure.^18^ Asthma medications were selected based on previous literature,^19^ primarily including bronchodilators (eg, albuterol), leukotriene modifiers (eg, montelukast), respiratory inhalant products (eg, fluticasone), nasal preparations (eg, fluticasone nasal), and adrenal cortical steroids (eg, budesonide) (eTable 1).

Asthma medications were systematically classified into pharmacologic groups based on their mechanisms of action and clinical roles: short-acting β2-agonists (SABA, eg, albuterol, terbutaline) for acute symptom relief; long-acting β2-agonists (LABA, eg, salmeterol, formoterol); inhaled corticosteroids (ICS) as anti-inflammatory agents (eg, fluticasone, budesonide); anticholinergics, subdivided into short-acting muscarinic antagonists (SAMA, eg, ipratropium) and long-acting muscarinic antagonists (LAMA, eg, tiotropium); leukotriene receptor antagonists (LTRA, eg, montelukast); mast cell stabilizers (eg, cromolyn); xanthine derivatives (eg, theophylline and combinations) as secondary bronchodilators; monoclonal antibodies (mAb, omalizumab, benralizumab); Antihistamines (eg, azelastine) and combination therapies (eg, ICS + LABA, triple ICS + LABA + LAMA).^20^^,^^21^

### Statistical analysis

All statistical analyses incorporated merged NHANES survey cycle sampling weights to adjust for nonresponse, survey noncoverage, and differential selection probabilities. Because NHANES field operations were suspended in March 2020 due to the COVID-19 pandemic, we followed NCHS analytic guidance for pandemic-affected cycles.^22^ The 2017–March 2020 pre-pandemic file was analyzed as a combined cycle using the NCHS-provided special weights and design variables, and 2019–March 2020 data were not analyzed separately. The 2021–2023 NHANES file was analyzed as a separate cycle using its corresponding survey weights. For pooled analyses across multiple NHANES cycles, survey weights were recalibrated according to the number of years represented by each cycle: standard 2-year cycles were assigned a factor of 2 divided by the total analytic period, whereas the 2017–March 2020 pre-pandemic cycle was assigned a factor of 3.2 divided by the total analytic period. All analyses incorporated the appropriate masked variance units, strata, and cycle-specific weights. Survey-weighted estimates with 95% confidence intervals (CIs) were calibrated to produce nationally representative results. Categorical variables were reported as frequency (survey-weighted percentage) and compared across groups using survey-weighted Pearson chi-square tests. Linear time trends in prevalence were quantified using linear regression models that included prevalence per cycle as the dependent variable and NHANES cycle as the continuous independent variable. Regression coefficients (β) with 95% CIs and corresponding *P*-values were derived to estimate the average percentage change in prevalence per cycle. Due to distinct shifts in asthma medication trends around 2007–2008, separate calculations were performed for 1999–2008 and 2009–2020. Statistical significance indicated significant increasing or decreasing temporal trends in prevalence. All statistical analyses were performed in R version 4.4.2 using the “survey” R package. Statistical significance was defined as a *P*-value <0.05.

## Results

### Participant characteristics and prevalence of different asthma statuses

Among 114,505 participants (median age 37 years; 51.2% male) in NHANES 1999–2023, the prevalence of lifetime and current asthma was 14.7% and 8.8%, respectively. Among individuals with current asthma, the prevalence of asthma attack and asthma-related emergency visits in past year was 49.9% and 18.0%, respectively (Table 1). Compared to males, females had higher asthma prevalence (lifetime: 15.6% vs. 13.8%; current: 10.1% vs. 7.4%) and higher prevalence of asthma attack (52.3% vs. 46.5%). In age stratification, lifetime asthma prevalence was 12.6% in 1–11 years old, peaked at 19.3% in 12–19 years old, then gradually declined (eTable 2). Current asthma prevalence was highest in 20–39 years (11.2%). The prevalence of asthma attack and asthma-related emergency visits in past year was highest in 1–11 years old and lowest in ≥60 years. Non-Hispanic Black had the highest lifetime asthma prevalence (17.8%), followed by other Hispanic (16.3%) (eTable 3). For current asthma prevalence, other race was highest (11.5%), followed by non-Hispanic White (9.0%). Non-Hispanic Black had the highest prevalence of asthma-related emergency visits in past year (28.9%), while non-Hispanic White had the lowest (13.5%) (eTable 3). Prevalence of asthma-related emergency visits in past year was highest in less than high school (24.1%) and lowest in college graduate or above (9.7%) (eTable 4). Economic level stratification showed asthma prevalence and disease severity among individuals with current asthma decreased with higher economic levels (eTable 5).

**Table 1:** Demographic characteristics and asthma status of participants stratified by sex.

Characteristics	Total (N = 114505)	Male (N = 56052, 48.8%)	Female (N = 58453, 51.2%)	*P*
Demographics				
Age group, n (%)				<0.001
1–11 years	28366 (14.9)	14402 (15.6)	13964 (14.2)	
12–19 years	19653 (11.2)	9954 (11.7)	9699 (10.7)	
20–39 years	22007 (27.5)	10203 (28.0)	11804 (27.1)	
40–59 years	20644 (27.3)	9922 (27.2)	10722 (27.3)	
60 years and over	23835 (19.2)	11571 (17.6)	12264 (20.7)	
Race, n (%)				<0.001
Mexican american	22670 (9.6)	11088 (10.2)	11582 (9.1)	
Other hispanic	9918 (6.6)	4691 (6.5)	5227 (6.7)	
Non-hispanic white	44061 (63.7)	21657 (63.9)	22404 (63.5)	
Non-hispanic black	26302 (12.0)	12944 (11.4)	13358 (12.6)	
Other race	11554 (8.1)	5672 (8.0)	5882 (8.1)	
Education level, n (%)				<0.001
Less than high school	16801 (16.5)	8355 (17.1)	8446 (15.9)	
High school	15367 (24.4)	7576 (25.3)	7791 (23.6)	
Some college or AA degree	18920 (30.5)	8359 (28.8)	10561 (32.1)	
College graduate or above	15253 (28.6)	7343 (28.8)	7910 (28.3)	
PIR category, n (%)				<0.001
<1.3	36910 (24.1)	17475 (22.6)	19435 (25.6)	
1.3–3.5	37914 (36.3)	18700 (35.9)	19214 (36.6)	
>3.5	27916 (39.6)	14199 (41.5)	13717 (37.8)	
Lifetime asthma, n (%)				<0.001
No	97789 (85.3)	48034 (86.2)	49755 (84.4)	
Yes	16716 (14.7)	8018 (13.8)	8698 (15.6)	
Current asthma^a^, n (%)				<0.001
No	95185 (91.2)	47000 (92.6)	48185 (89.9)	
Yes	9559 (8.8)	4282 (7.4)	5277 (10.1)	
Asthma attack in past year among individuals with current asthma^a^, n (%)				<0.001
No	4749 (50.1)	2217 (53.5)	2532 (47.7)	
Yes	4790 (49.9)	2056 (46.5)	2734 (52.3)	
Asthma-related emergency visits in past year among individuals with current asthma^a^, n (%)				0.180
No	5784 (82.0)	2518 (82.9)	3266 (81.4)	
Yes	1775 (18.0)	823 (17.1)	952 (18.6)	
Asthma medication use among asthma population^b^, n (%)				0.003
No	4129 (54.4)	1664 (51.7)	2465 (56.2)	
Yes	4336 (45.6)	2027 (48.3)	2309 (43.8)	

All categorical variables were presented as frequency (survey-weighted percentage). Difference was tested using the survey-weighted Chi-square tests.

Abbreviations: PIR, poverty-to-income ratio.

a Due to inconsistencies in the question routing logic for three asthma-related items (current asthma, asthma attack in past year, and asthma-related emergency visits in past year) between the 1999–2000 cycle and other cycles, data from the 1999–2000 cycle were not used for these three asthma status variables.

b Since NHANES 2021–2023 has not yet released detailed prescription medication data, the analysis of medication use among the asthma population is limited to prescription drug information from 1999 to 2020.

### Trends in prevalence of different asthma statuses

**Fig. 1:**
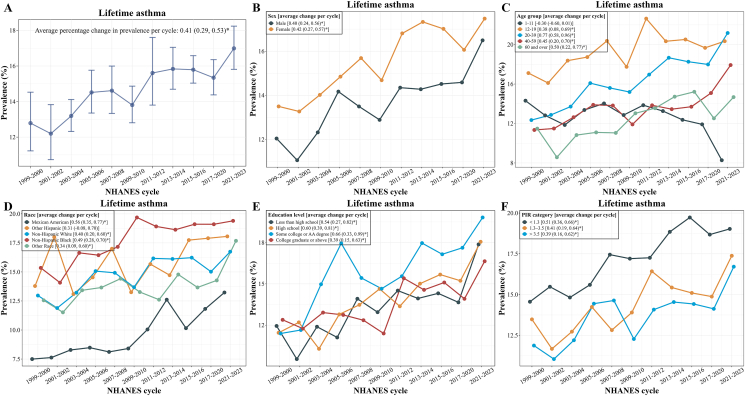
**Trends in lifetime asthma prevalence in the general population and across demographic subgroups.** (A) Overall population; (B) Sex; (C) Age; (D) Race; (E) Education level; (F) PIR. The points on the line graph represent the survey-weighted prevalence rate for each cycle. The error bars indicate the 95% CI for weighted prevalence. The estimate (β) and 95% CI in corresponding figure legends were obtained from linear regression models that included prevalence per cycle as the dependent variable and NHANES cycle as the continuous independent variable. The estimate (β) represents the average percentage change in prevalence per cycle. Asterisks indicate significant estimates, showing statistically significant increasing or decreasing temporal trends in prevalence. Abbreviations: NHANES, National Health and Nutrition Examination Survey; PIR, poverty-to-income ratio.

Over the past 2 decades, both lifetime and current asthma prevalence have increased significantly. Lifetime asthma prevalence rose from 11.2% (1999–2000) to 15.8% (2021–2023), with an average significant increase of 0.41% per cycle (Fig. 1A; Table 2). Current asthma prevalence increased from 6.5% (2001–2002) to 9.3% (2021–2023), with an average significant increase of 0.24% per cycle (eFigure 1A; Table 2). Among individuals with current asthma, the prevalence of asthma attacks and asthma-related emergency visits in past year declined significantly, decreasing by 0.99% and 1.94% per cycle, respectively (eFigure 2A; eFigure 3A; Table 2). The prevalence of asthma-related emergency visits decreased from 24.5% (2001–2002) to 9.1% (2021–2023).

**Table 2:** Prevalence of different asthma statuses across cycles in the general population and sex-stratified subgroups.

Cycle	Lifetime asthma (N = 114505)	Current asthma^a^ (N = 104744)	Current asthma	
			Asthma attack in past year^a^ (N = 9539)	Asthma-related emergency visits in past year^a^ (N = 7559)
Total (N = 114505)	14.7 (14.4, 15.1)	8.8 (8.5, 9.1)	49.9 (48.4, 51.4)	18.0 (16.7, 19.3)
1999–2000 (N = 9484)	11.2 (12.8, 14.5)	–	–	–
2001–2002 (N = 10460)	10.7 (12.2, 13.8)	6.5 (7.6, 8.9)	50.5 (56.0, 61.3)	24.5 (28.9, 33.9)
2003–2004 (N = 9626)	12.3 (13.2, 14.1)	7.1 (7.9, 8.9)	44.7 (49.2, 53.7)	17.9 (23.6, 30.6)
2005–2006 (N = 9809)	13.4 (14.5, 15.8)	7.8 (8.7, 9.7)	55.2 (59.5, 63.7)	20.8 (27.0, 34.3)
2007–2008 (N = 9659)	13.3 (14.6, 16.0)	7.2 (8.3, 9.6)	47.4 (52.2, 57.0)	19.4 (25.9, 33.7)
2009–2010 (N = 10092)	12.8 (13.8, 14.9)	6.9 (7.6, 8.5)	41.2 (46.2, 51.3)	24.2 (30.7, 38.1)
2011–2012 (N = 9354)	13.8 (15.6, 17.6)	7.7 (9.1, 10.7)	47.5 (52.5, 57.5)	11.6 (16.0, 21.6)
2013–2014 (N = 9760)	14.7 (15.8, 17.1)	8.6 (9.4, 10.3)	43.8 (47.6, 51.4)	12.4 (15.0, 18.1)
2015–2016 (N = 9564)	15.0 (15.8, 16.6)	8.5 (9.3, 10.1)	44.2 (48.7, 53.2)	11.3 (14.5, 18.4)
2017–2020 (N = 14969)	14.4 (15.3, 16.4)	8.0 (8.8, 9.6)	39.0 (44.2, 49.5)	12.7 (15.3, 18.4)
2021–2023 (N = 11728)	15.8 (17.0, 18.2)	9.3 (10.4, 11.7)	44.1 (48.6, 53.1)	9.1 (12.0, 15.6)
Average change per cycle^b^	**0.41 (0.28, 0.54)**	**0.24 (0.09, 0.38)**	**−0.99 (−1.94, −0.03)**	**−1.94 (−2.96, −0.93)**
*P*-value for trend^b^	**<0.001**	**0.005**	**0.044**	**0.002**
Male				
Total (N = 56052)	13.8 (13.3, 14.2)	7.4 (7.0, 7.7)	46.5 (44.1, 48.9)	17.1 (15.5, 18.8)
1999–2000 (N = 4629)	10.4 (12.0, 14.0)	–	–	–
2001–2002 (N = 5047)	9.5 (11.1, 12.8)	5.6 (6.7, 7.9)	44.9 (54.1, 63.0)	20.2 (27.2, 35.6)
2003–2004 (N = 4707)	11.3 (12.3, 13.4)	5.9 (6.7, 7.6)	39.4 (45.5, 51.7)	17.6 (25.9, 36.5)
2005–2006 (N = 4781)	12.3 (14.2, 16.2)	6.4 (7.6, 9.0)	53.5 (61.5, 68.9)	19.8 (28.9, 40.1)
2007–2008 (N = 4844)	12.2 (13.5, 14.9)	5.6 (7.0, 8.7)	41.8 (51.5, 61.1)	19.5 (25.5, 32.7)
2009–2010 (N = 5010)	11.6 (12.9, 14.3)	5.6 (6.5, 7.5)	34.1 (44.5, 55.3)	23.4 (30.4, 38.5)
2011–2012 (N = 4655)	12.6 (14.3, 16.3)	6.7 (8.2, 10.0)	42.8 (50.4, 58.0)	8.2 (11.8, 16.6)
2013–2014 (N = 4797)	12.8 (14.3, 15.9)	6.4 (7.4, 8.6)	35.4 (40.5, 45.9)	9.9 (13.6, 18.4)
2015–2016 (N = 4700)	13.0 (14.5, 16.2)	6.5 (7.3, 8.2)	36.0 (42.7, 49.8)	10.3 (14.1, 19.0)
2017–2020 (N = 7420)	13.0 (14.6, 16.4)	6.2 (7.0, 7.9)	35.1 (41.4, 48.0)	11.3 (14.3, 17.9)
2021–2023 (N = 5462)	15.2 (16.5, 17.9)	8.1 (9.3, 10.5)	32.8 (39.6, 46.8)	8.3 (11.6, 16.0)
Average change per cycle^b^	**0.40 (0.24, 0.56)**	0.17 (−0.02, 0.35)	**−1.70 (−2.99, −0.40)**	**−2.13 (−3.33, −0.93)**
*P*-value for trend^b^	**<0.001**	0.164	**0.017**	**0.004**
Female				
Total (N = 58453)	15.6 (15.1, 16.1)	10.1 (9.7, 10.5)	52.3 (50.4, 54.2)	18.6 (16.9, 20.4)
1999–2000 (N = 4855)	11.8 (13.5, 15.3)	–	–	–
2001–2002 (N = 5413)	11.5 (13.3, 15.2)	7.0 (8.5, 10.2)	50.4 (57.4, 64.1)	22.4 (30.1, 39.2)
2003–2004 (N = 4919)	12.6 (14.0, 15.6)	8.0 (9.2, 10.4)	44.3 (51.8, 59.1)	14.3 (22.3, 32.9)
2005–2006 (N = 5028)	13.7 (14.8, 16.0)	8.6 (9.8, 11.1)	52.9 (58.1, 63.1)	16.5 (25.5, 37.1)
2007–2008 (N = 4815)	14.0 (15.7, 17.5)	8.3 (9.6, 11.0)	47.0 (52.7, 58.4)	16.7 (26.2, 38.6)
2009–2010 (N = 5082)	13.5 (14.7, 16.0)	7.9 (8.8, 9.8)	42.6 (47.5, 52.4)	22.6 (30.9, 40.6)
2011–2012 (N = 4699)	14.5 (16.8, 19.3)	8.5 (10.0, 11.8)	47.6 (54.2, 60.6)	13.0 (19.3, 27.8)
2013–2014 (N = 4963)	15.8 (17.3, 19.0)	10.1 (11.3, 12.7)	46.4 (52.0, 57.5)	11.7 (15.9, 21.3)
2015–2016 (N = 4864)	15.3 (17.0, 18.8)	9.5 (11.1, 13.0)	45.8 (52.5, 59.1)	11.1 (14.8, 19.4)
2017–2020 (N = 7549)	14.3 (16.1, 17.9)	9.1 (10.4, 11.9)	40.0 (46.0, 52.1)	12.9 (16.0, 19.5)
2021–2023 (N = 6266)	16.3 (17.5, 18.7)	10.3 (11.6, 12.9)	50.4 (55.6, 60.7)	9.3 (12.3, 16.1)
Average change per cycle^b^	**0.42 (0.27, 0.57)**	**0.30 (0.15, 0.46)**	−0.48 (−1.45, 0.49)	**−1.82 (−2.82, −0.83)**
*P*-value for trend^b^	**<0.001**	**0.002**	0.283	**0.003**

All categorical variables were presented as frequency (survey-weighted percentage).

a Due to inconsistencies in the question routing logic for three asthma-related items (current asthma, asthma attack in past year, and asthma-related emergency visits in past year) between the 1999–2000 cycle and other cycles, data from the 1999–2000 cycle were not used for these three asthma status variables.

b The estimate (*β*), 95% CI, and *P*-value for trend were obtained from linear regression models that included prevalence per cycle as the dependent variable and NHANES cycle as the continuous independent variable. The estimate (*β*) represents the average percentage change in prevalence per cycle. Bold indicates significant estimates, showing statistically significant increasing or decreasing temporal trends in prevalence.

In stratified analyses of trends in lifetime asthma prevalence, males and females showed comparable significant increases (Fig. 1B; Table 2). Only the 1–11 age group and other Hispanic ethnicity showed non-significant trends, while all other demographic strata increased significantly (Fig. 1C–F; eTable6-S9). The 1–11 age group showed a borderline significant decreasing trend (Fig. 1C; eTable6). In the education level and economic level groups, the lowest per-cycle average increase for lifetime asthma prevalence were observed in the highest education level and economic level groups (Fig. 1E and F; eTable8-S9).

Current asthma prevalence increased significantly in females but not males (eFig. 1B–Table 2). Regarding age stratification, only the 20–39 and ≥ 60-year groups exhibited significant increasing trends (eFigure1C, eTable6). The 1-11-year-old group exhibited the only non-significant downward trend. Racial stratification revealed that only other Hispanic individuals lacked a significant trend, while Black individuals showed the largest significant increase (eFigure1D, eTable7). Education level and economic level stratification showed that only the highest education level and highest economic level groups maintained stable prevalence across cycles with no significant trend changes (eFigure1E-F, eTable8-S9).

Among individuals with current asthma, asthma attack prevalence decreased significantly in males (eFig. 2B–Table 2). The ≥60-year group exhibited the only significant increase (eFigure2C, eTable6). No significant trends were observed across racial groups (eFigure2D, eTable7). Stratification by education level revealed a significant decrease only among college graduates or above (eFigure2E, eTable8). While no economic level groups showed significant trend changes, higher economic levels were associated with progressively larger decreasing trends (eFigure2F, eTable 9).

The groups with the greatest decline in the prevalence of asthma-related emergency visits across stratifications mainly came from: male, those aged ≥60 years, other Hispanic, and the lowest education and economic level populations (eFigure 3, Table 2, eTable6-S9).

### Trends in prevalence of asthma medication use

**Fig. 2:**
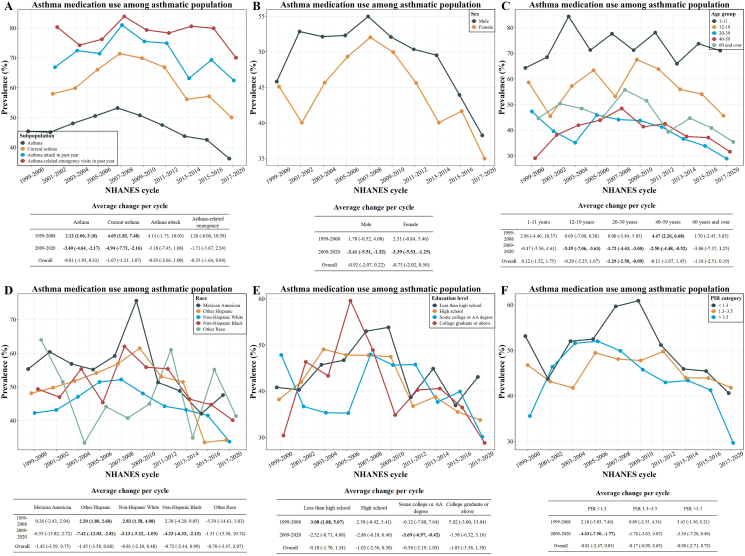
**Trends in prevalence of asthma medication use among asthmatic population and across demographic subgroups.** (A) Trends in prevalence of asthma medication use among populations with different asthma statuses; (B–F) Trends in prevalence of asthma medication use among asthmatic population stratified by (B) sex; (C) age; (D) race; (E) education level; (F) PIR. The points on the line graph represent the survey-weighted prevalence rate for each cycle. At the bottom of each panel: The estimate (β) and 95% CI in corresponding figure legends were obtained from linear regression models that included prevalence per cycle as the dependent variable and NHANES cycle as the continuous independent variable. The estimate (β) represents the average percentage change in prevalence per cycle. Due to distinct shifts in asthma medication trends around 2007–2008, separate calculations were performed for 1999–2008 and 2009–2020. Bold indicates significant estimates, showing statistically significant increasing or decreasing temporal trends in prevalence. Abbreviations: NHANES, National Health and Nutrition Examination Survey; PIR, poverty-to-income ratio.

The prevalence of asthma medication use was 45.6% (95% CI: 44.0%, 47.1%) in those with lifetime asthma, 61.8% (95% CI: 59.9%, 63.6%) in those with current asthma, 71.0% (95% CI: 68.7%, 73.1%) in those experiencing asthma attacks within the past year, and 78.1% (95% CI: 74.6%, 81.2%) in those with asthma-related emergency visits within the past year (Table 3, Fig. 2A). In all asthma subpopulations, the prevalence of asthma medication use increased from 1999 to 2000 to a peak in 2007–2008, then decreased, with no significant average change across all cycles. Segmented analysis showed significant increasing followed by decreasing trends on both sides of 2007–2008 in those with lifetime and current asthma.

**Table 3:** Prevalence of asthma medication use among populations with different asthma statuses across cycles.

Cycle	Asthma medication use among subpopulation			
	Lifetime asthma (N = 8465)	Current asthma^a^ (N = 5669)	Asthma attack in past year^a^ (N = 3175)	Asthma-related emergency visits in past year^a^ (N = 1225)
Total (N = 8465)	45.6 (44.0, 47.1)	61.8 (59.9, 63.6)	71.0 (68.7, 73.1)	78.1 (74.6, 81.2)
1999–2000 (N = 613)	39.6 (45.4, 51.3)	–	–	–
2001–2002 (N = 698)	40.4 (45.1, 49.9)	52.9 (58.0, 62.9)	60.9 (66.9, 72.4)	68.0 (80.4, 88.7)
2003–2004 (N = 721)	43.8 (48.1, 52.5)	56.5 (59.9, 63.2)	64.5 (72.5, 79.2)	60.8 (74.3, 84.3)
2005–2006 (N = 775)	44.9 (50.6, 56.3)	60.0 (66.1, 71.6)	64.9 (71.4, 77.2)	63.2 (76.3, 85.8)
2007–2008 (N = 846)	47.8 (53.2, 58.6)	65.5 (71.4, 76.7)	73.0 (81.0, 87.1)	70.5 (83.9, 91.9)
Average change per cycle^b^ (1999–2008)	**2.12 (1.06, 3.18)**	**4.65 (1.82, 7.48)**	4.14 (−1.75, 10.03)	1.26 (−8.06, 10.59)
*P*-value for trend^b^	**0.008**	**0.019**	0.094	0.619
2009–2010 (N = 891)	46.4 (50.8, 55.2)	65.1 (69.9, 74.3)	69.0 (75.6, 81.1)	67.6 (79.4, 87.7)
2011–2012 (N = 860)	41.5 (47.5, 53.6)	60.2 (66.9, 72.9)	67.5 (75.0, 81.2)	65.6 (78.4, 87.4)
2013–2014 (N = 892)	39.1 (43.8, 48.5)	49.7 (56.2, 62.4)	54.6 (63.2, 71.0)	72.6 (80.6, 86.7)
2015–2016 (N = 836)	38.2 (42.5, 47.0)	52.9 (57.1, 61.3)	64.3 (69.3, 73.9)	66.2 (80.0, 89.0)
2017–2020 (N = 1333)	32.3 (36.3, 40.5)	43.8 (50.1, 56.4)	54.1 (62.5, 70.1)	58.9 (70.0, 79.2)
Average change per cycle^b^ (2009–2020)	**−3.40 (−4.64, −2.17)**	**−4.94 (−7.71, −2.16)**	−3.18 (−7.45, 1.08)	−1.71 (−5.67, 2.24)
*P*-value for trend^b^	**0.003**	**0.011**	0.098	0.262
Average change per cycle^b^	−0.81 (−1.95, 0.33)	−1.07 (−3.21, 1.07)	−0.83 (−2.66, 1.00)	−0.35 (−1.64, 0.94)
*P*-value for trend^b^	0.141	0.275	0.321	0.540

All prevalences and 95% CI were survey-weighted.

Bold indicates significant estimates, showing statistically significant increasing or decreasing temporal trends in prevalence.

a Due to inconsistencies in the question routing logic for three asthma-related items (current asthma, asthma attack in past year, and asthma-related emergency visits in past year) between the 1999–2000 cycle and other cycles, data from the 1999–2000 cycle were not used for these three asthma status variables.

b The estimate (*β*), 95% CI, and *P*-value for trend were obtained from linear regression models that included prevalence per cycle as the dependent variable and NHANES cycle as the continuous independent variable. The estimate (*β*) represents the average percentage change in prevalence per cycle.

Males (48.3%) had a higher prevalence of asthma medication use than females (43.8%) (Table 1). The highest prevalence was observed among children aged 1–11 (73.0%) (eTable2). Mexican Americans had the highest prevalence (52.8%) (eTable3). The prevalence of asthma medication uses from low to high education level are 45.0%, 40.8%, 38.6%, and 39.6% (eTable4). The prevalence from low to high economic level are 50.2%, 45.5%, and 43.1% (eTable5). This indicates that lower education and economic levels have higher prevalence of asthma medication use. Demographic stratification analyses also showed consistent trends, with the significant trends on both sides of 2007–2008 mainly concentrated in the significant decreasing trend from 2009 to 2020 (Fig. 2B–F).

**Fig. 3:**
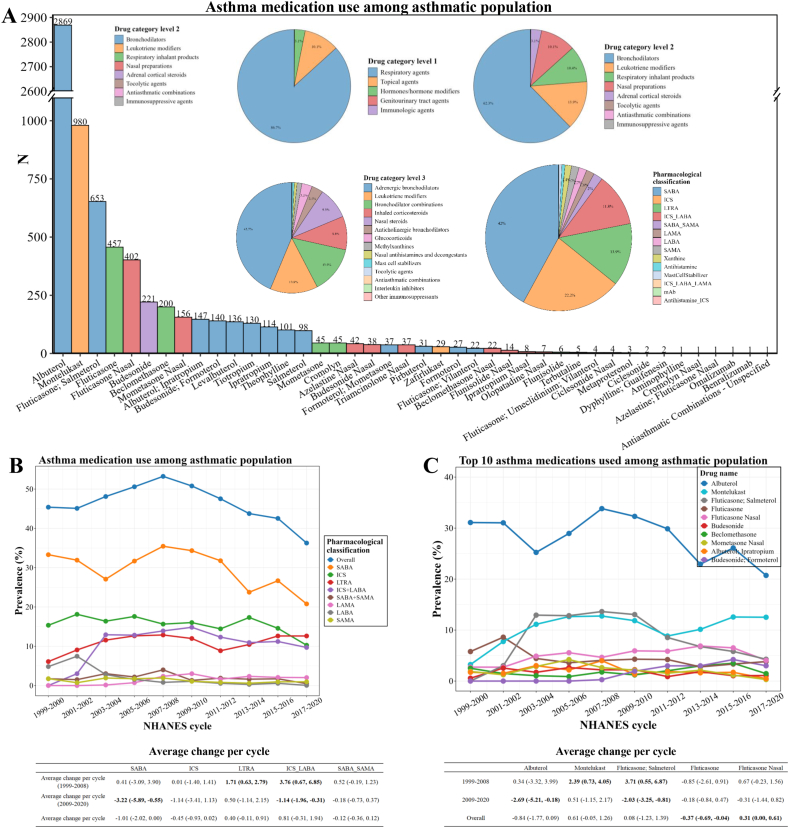
**Asthma medication use among asthmatic population.** (A) Number of users for each medication, and the proportion of users accounted for by different medication classifications (three-level therapeutic categories from the Multum Lexicon database and pharmacological classifications) in total medication utilization instances. (B) Trends in the prevalence of asthma medication use by pharmacological classifications. (C) Trends in the prevalence of top 10 most commonly used asthma medications. The points on the line graph represent the survey-weighted prevalence rate for each cycle. At the bottom of panels B and C: The estimate (β) and 95% CI were obtained from linear regression models that included prevalence per cycle as the dependent variable and NHANES cycle as the continuous independent variable. The estimate (β) represents the average percentage change in prevalence per cycle. Due to distinct shifts in asthma medication trends around 2007–2008, separate calculations were performed for 1999–2008 and 2009–2020. Bold indicates significant estimates, showing statistically significant increasing or decreasing temporal trends in prevalence. Abbreviations: NHANES, National Health and Nutrition Examination Survey; SABA, short-acting β2-agonists; LABA, long-acting β2-agonists; ICS, inhaled corticosteroids; SAMA, short-acting muscarinic antagonists; LAMA, long-acting muscarinic antagonists; LTRA, leukotriene receptor antagonists.

Asthma medication classifications among those with lifetime asthma are shown in Fig. 3A. The top 5 medications were Albuterol (39.6%), Montelukast (13.5%), Fluticasone; Salmeterol (9.0%), Fluticasone (6.3%), and Fluticasone Nasal (5.6%), accounting for 74.0% of total medication usage. The usage trends for most medications and their classifications resembled overall medication patterns (Tables S10–S14; Fig. 3B–C). The increasing trend in overall medication usage from 1999 to 2008 was mainly driven by Montelukast (prevalence increased from 2.0% to 9.3%; eTable 14) and its medication classifications (second-level: leukotriene modifiers; pharmacological: LTRA), and the combination drug Fluticasone/Salmeterol (prevalence increased from 0.0% to 11.2%; eTable14) with its classifications (third-level: bronchodilator combinations; pharmacological: ICS + LABA).

The significant decreasing trend from 2009 to 2020 was primarily attributed to Albuterol (prevalence decreased from 28.5% to 17.9%; eTable 14) and its related medication categories (first-level: respiratory agents; second-level: bronchodilators; third-level: adrenergic bronchodilators; pharmacological: SABA). Additionally, the combination drug Fluticasone/Salmeterol (prevalence decreased from 10.4% to 2.8%; eTable14) and its classifications (third-level: bronchodilator combinations; pharmacological: ICS + LABA) also showed significant decreases during 2009–2020. Notably, another combination drug for acute asthma attacks, Albuterol/Ipratropium (SABA + SAMA), showed no utilization during 1999–2006, but demonstrated an increasing usage trend starting from 2007, reaching 3.0% in 2017–2020 (eTable14).

## Discussion

Across NHANES cycles, the prevalence of self-reported asthma attacks and asthma-related emergency visits in the past year declined among participants with current asthma. The prevalence of asthma medication uses among asthmatic individuals showed a significant increase during 1999–2008, followed by a decrease between 2009 and 2020. This shift may be attributed to evolving treatment paradigms, the adoption of alternative therapeutic approaches, and changes in healthcare policies. These findings illuminate the dynamic trends in asthma epidemiology and the transformation of treatment strategies, providing valuable insights for future precision-based prevention and management approaches.

This study reveals that both lifetime and current asthma prevalence in the United States increased steadily between 1999 and 2023, consistent with previous reports from the Behavioral Risk Factor Surveillance System (BRFSS),^23^ and the National Health Interview Survey (NHIS).^24^ These findings underscore that asthma remains a major public health concern in the United States. Several factors may underlie this upward trajectory, including escalating environmental pollution, greater diversity and intensity of aeroallergen exposure, and lifestyle changes associated with urbanization^25^, ^26^, ^27^. Enhanced disease awareness and the refinement of diagnostic criteria have likely led to improved case ascertainment, thereby contributing to the apparent rise in prevalence.^28^ In addition, the parallel increase in national obesity rates may play a pivotal role.^29^ Obesity has been well established as an independent risk factor for asthma development and progression. Excess adiposity may exacerbate asthma via immune dysregulation, heightened systemic inflammation, and compromised airway mechanics.^30^ Lifestyle shifts toward higher fast-food consumption and insufficient physical activity have almost certainly fueled the obesity epidemic and, by extension, asthma risk.^31^

The decline in asthma attacks and asthma-related emergency visits occurred during a period of major changes in asthma care, including greater emphasis on controller-based management and broader use of stepwise treatment strategies following GINA and other guideline updates,^32^^,^^33^ expansion of severe-asthma therapies,^34^ and changes in insurance coverage and healthcare delivery after implementation of the Affordable Care Act.^35^ Increased attention to asthma action plans and self-management tools may also have contributed to changes in asthma care during this period.^36^ However, these factors were not directly measured in the present NHANES analysis and should be interpreted as possible contextual explanations rather than causal mechanisms established by our data. Future studies linking survey data with clinical, pharmacy, insurance, and healthcare-utilization records are needed to clarify the drivers of national trends in asthma morbidity.

Consistent with previous studies,^6^^,^^23^^,^^37^^,^^38^ this study identifies significant sociodemographic disparities in asthma prevalence, with higher prevalence observed among women, non-Hispanic Black individuals, and those with lower socioeconomic status. Demographic shifts, including a growing proportion of females and non-Hispanic Black residents, may partly explain the national rise in prevalence and could further entrench health inequities.^39^ The higher prevalence of asthma in women may be related to hormonal fluctuations, increased airway inflammation, and heightened sensitivity of the immune system. Higher prevalence of asthma attack and asthma-related emergency visits among non-Hispanic Black and economically disadvantaged groups likely reflect their greater exposure to traffic and industrial pollution, residential crowding, allergen accumulation, and limited healthcare access.^40^^,^^41^

The highest cross-sectional prevalence of asthma attacks and emergency visits was observed in children aged 1–11 years. However, repeated cross-sectional estimates showed decreasing trends in lifetime and current asthma prevalence in this age group. These findings suggest that childhood asthma remains an important disease burden, although population-level improvements in prevention and management may have occurred. Early childhood asthma management has benefitted from advances in screening technologies and the implementation of preventive measures, such as reducing exposure to environmental triggers and improving air quality.^42^^,^^43^ However, a report from the CDC in the United States indicates that in 2013, only half of children with asthma received an asthma action plan, and fewer than half received recommendations for environmental control.^44^ These results highlight the need for further improvement in the prevention and management of childhood asthma. Adolescents (12–19 years old) exhibit the highest cross-sectional lifetime asthma prevalence and the most significant decline in asthma attack prevalence longitudinally. The increased asthma prevalence in adolescents may be attributed to the hormonal changes during puberty, which can exacerbate airway inflammation and immune system sensitivity.^45^^,^^46^ The observed decline in asthma attacks among adolescents across NHANES cycles may reflect population-level changes in asthma management, healthcare access, and patient education, although these factors could not be directly evaluated in this study. Additionally, lifestyle factors such as increased physical activity and exposure to environmental pollutants may initially elevate asthma prevalence but may become more manageable with appropriate interventions over time.^47^ The greatest population-level declines in asthma-related emergency visits were observed among older adults. Potential explanations may include changes in chronic-disease management, healthcare access, or medication use, although these factors were not directly assessed in the present analysis.^48^

Our study shows that asthma medication use among individuals with asthma increased from 1999 to 2008 and then declined between 2009 and 2020, a pattern most evident for adrenergic bronchodilators and fixed-dose combination therapies. The early increase may reflect changes in asthma awareness, medication availability, and treatment practices during that period. Prescription prevalence of albuterol and fluticasone/salmeterol decreased substantially after 2009, during a period when asthma management increasingly emphasized symptom-stratified, stepwise care and the use of the lowest effective controller regimen.^49^ In addition, biologic therapies for severe uncontrolled asthma and SMART/MART or anti-inflammatory reliever strategies using ICS-formoterol have become important developments in asthma treatment.^50^^,^^51^ However, NHANES does not provide sufficient treatment-specific information to determine whether these medication trends were driven by guideline implementation, changes in prescribing behavior, medication access, adherence, biologic therapies, SMART/MART, or evolving asthma severity.

This study found that medication usage prevalence were highest among children and adolescents. First, children and adolescents are the age groups most affected by asthma, with symptoms often being more pronounced and frequent, thus requiring long-term medication management.^52^ For younger children, asthma monitoring and management rely heavily on parental and healthcare system support, making medication use more widespread.^53^ In contrast, adults, particularly those from low-income groups, may experience medication interruptions.^54^ Medication usage prevalence are highest among minority populations and those in the lowest socioeconomic groups, likely due to poorer asthma control, leading to greater reliance on pharmacological treatment. These groups often depend more on healthcare subsidies and public health programs, with medication being a key means of managing chronic conditions such as asthma. They may seek medical help more frequently and use medication to control disease symptoms. Data From the National Asthma Survey showed that 26% of black children and 19% of Hispanic children reported receiving a daily dose of a short-acting β-agonist compared with 12% of white children.^55^ Furthermore, living conditions in low-income households are often poor, with factors such as poor air quality, abundant allergens, and limited access to healthcare services, further contributing to their reliance on medication for asthma management.^56^

Several limitations should be acknowledged. First, the cross-sectional NHANES design precludes causal inference. Second, asthma diagnosis, attack history, and asthma-related emergency visits were self-reported, introducing potential recall bias. Third, we could not fully account for external influences, such as changes in healthcare delivery systems, public-health education campaigns, or the introduction of new therapies, that may have independently affected disease trends. Fourth, this study could not separately evaluate biologic therapies, SMART/MART, or anti-inflammatory reliever strategies because detailed treatment-specific information was unavailable. Fourth, this study could not separately evaluate biologic therapies, SMART/MART, or anti-inflammatory reliever strategies because detailed treatment-specific information was unavailable. Fifth, the COVID-19 pandemic may have influenced NHANES data collection, sampling procedures, and participant response patterns, potentially affecting comparability across cycles. Although we used the NCHS-recommended survey weights for the 2017–March 2020 and 2021–2023 cycles, residual pandemic-related differences cannot be fully excluded. Sixth, because NHANES is a repeated cross-sectional survey rather than a longitudinal cohort, our findings reflect population-level temporal trends across survey cycles and cannot be interpreted as within-person changes in asthma onset, persistence, treatment response, or disease progression.

In summary, from 1999 to 2023 the United States experienced continued increases in lifetime and current asthma prevalence, even as prevalence of asthma attack and asthma-related emergency visit among asthmatic individuals declined, evidence of improving disease management. Temporal changes in medication use mirror evolving clinical guidelines and reimbursement policies. Strengthening environmental and social interventions for high-risk groups and optimizing evidence-based pharmacotherapy will be essential to further reduce the national asthma burden.

## Use of generative artificial intelligence (AI) and AI-assisted technologies

Nothing to disclose.

## CRediT author statement

Leiwen Fu: Conceptualization, Methodology, Formal analysis, Funding acquisition, Writing - Original Draft, Writing - Review & Editing.

Ke Liu: Validation, Writing - Original Draft, Writing - Review & Editing.

Yuxia Du: Validation, Writing - Review & Editing.

Jian Du: Funding acquisition, Writing - Review & Editing.

Liang Li: Funding acquisition, Writing - Review & Editing.

Yang Liu: Methodology, Writing - Review & Editing.

Weitao Su: Conceptualization, Formal analysis, Funding acquisition, Writing - Original Draft, Writing - Review & Editing.

## Ethics approval

The NHANES protocol was approved by the NCHS Research Ethics Review Board, and informed consent was obtained from all participants.

## Data availability statement

The datasets generated and/or analyzed during the current study are available in the National Health and Nutrition Examination Survey repository, [https://wwwn.cdc.gov/nchs/nhanes/continuousnhanes/default.aspx].

## Funding source

This study was supported by the National Natural Science Foundation of China (NO. 82504532); the Young and Middle-aged Talent Development Program of Beijing Chest Hospital (ZQNGG202604); Beijing Municipal Health Commission (2022-2-010 & 2024-03-11); Institutional Fund of The Second Affiliated Hospital of Fujian Medical University (BS202503). All funding parties did not have any role in the design of the study or in the explanation of the data.

## Conflict of interest

All authors declare no conflict of interest.

## Footnotes

Appendix A Supplementary data to this article can be found online at https://doi.org/10.1016/j.waojou.2026.101426.
