# Supplementary material for: The shifting landscape of asthma in the United States: An over 20-year analysis of prevalence, severity, and medication paradigms, 1999–2023
Source: World Allergy Organ J. 2026 Jul 2;19(8):101426. doi: 10.1016/j.waojou.2026.101426 (PMC13352078; doi:10.1016/j.waojou.2026.101426)
Supplement: Multimedia component 1 [file mmc1.docx]

**eTable 1** **Description of selected prescription drugs for asthma**

| Drug code | Drug name | N | Drug category level 1 | Drug category level 2 | Drug category level 3 | Pharmacological classification |
| --- | --- | --- | --- | --- | --- | --- |
| d00749 | Albuterol | 2869 | Respiratory Agents | Bronchodilators | Adrenergic Bronchodilators | SABA |
| d04289 | Montelukast | 980 | Respiratory Agents | Leukotriene Modifiers | - | LTRA |
| d04611 | Fluticasone; Salmeterol | 653 | Respiratory Agents | Bronchodilators | Bronchodilator Combinations | ICS+LABA |
| d01296 | Fluticasone | 457 | Respiratory Agents | Respiratory Inhalant Products | Inhaled Corticosteroids | ICS |
| d04283 | Fluticasone Nasal | 402 | Topical Agents | Nasal Preparations | Nasal Steroids | ICS |
| d04276 | Budesonide | 221 | Hormones/Hormone Modifiers | Adrenal Cortical Steroids | Glucocorticoids | ICS |
| d00760 | Beclomethasone | 200 | Respiratory Agents | Respiratory Inhalant Products | Inhaled Corticosteroids | ICS |
| d04223 | Mometasone Nasal | 156 | Topical Agents | Nasal Preparations | Nasal Steroids | ICS |
| d04066 | Albuterol; Ipratropium | 147 | Respiratory Agents | Bronchodilators | Bronchodilator Combinations | SABA+SAMA |
| d04795 | Budesonide; Formoterol | 140 | Respiratory Agents | Bronchodilators | Bronchodilator Combinations | ICS+LABA |
| d04427 | Levalbuterol | 136 | Respiratory Agents | Bronchodilators | Adrenergic Bronchodilators | SABA |
| d04829 | Tiotropium | 130 | Respiratory Agents | Bronchodilators | Anticholinergic Bronchodilators | LAMA |
| d00265 | Ipratropium | 114 | Respiratory Agents | Bronchodilators | Anticholinergic Bronchodilators | SAMA |
| d00142 | Theophylline | 101 | Respiratory Agents | Bronchodilators | Methylxanthines | Xanthine |
| d03759 | Salmeterol | 98 | Respiratory Agents | Bronchodilators | Adrenergic Bronchodilators | LABA |
| d05262 | Mometasone | 45 | Respiratory Agents | Respiratory Inhalant Products | Inhaled Corticosteroids | ICS |
| d00200 | Cromolyn | 45 | Respiratory Agents | Respiratory Inhalant Products | Mast Cell Stabilizers | Mast Cell Stabilizer |
| d04068 | Azelastine Nasal | 42 | Topical Agents | Nasal Preparations | Nasal Antihistamines And Decongestants | Antihistamine |
| d03640 | Budesonide Nasal | 38 | Topical Agents | Nasal Preparations | Nasal Steroids | ICS |
| d07660 | Formoterol; Mometasone | 37 | Respiratory Agents | Bronchodilators | Bronchodilator Combinations | ICS+LABA |
| d04233 | Triamcinolone Nasal | 37 | Topical Agents | Nasal Preparations | Nasal Steroids | ICS |
| d00755 | Pirbuterol | 31 | Respiratory Agents | Bronchodilators | Adrenergic Bronchodilators | SABA |
| d04053 | Zafirlukast | 29 | Respiratory Agents | Leukotriene Modifiers | - | LTRA |
| d04572 | Formoterol | 27 | Respiratory Agents | Bronchodilators | Adrenergic Bronchodilators | LABA |
| d08100 | Fluticasone; Vilanterol | 22 | Respiratory Agents | Bronchodilators | Bronchodilator Combinations | ICS+LABA |
| d04275 | Beclomethasone Nasal | 22 | Topical Agents | Nasal Preparations | Nasal Steroids | ICS |
| d04279 | Flunisolide Nasal | 14 | Topical Agents | Nasal Preparations | Nasal Steroids | ICS |
| d04235 | Ipratropium Nasal | 8 | Topical Agents | Nasal Preparations | Nasal Antihistamines And Decongestants | SAMA |
| d07132 | Olopatadine Nasal | 7 | Topical Agents | Nasal Preparations | Nasal Antihistamines And Decongestants | Antihistamine |
| d00761 | Flunisolide | 6 | Respiratory Agents | Respiratory Inhalant Products | Inhaled Corticosteroids | ICS |
| d00752 | Terbutaline | 5 | Genitourinary Tract Agents | Tocolytic Agents | - | SABA |
| d08666 | Fluticasone; Umeclidinium; Vilanterol | 4 | Respiratory Agents | Bronchodilators | Bronchodilator Combinations | ICS+LABA+LAMA |
| d05899 | Ciclesonide Nasal | 4 | Topical Agents | Nasal Preparations | Nasal Steroids | ICS |
| d00750 | Metaproterenol | 3 | Respiratory Agents | Bronchodilators | Adrenergic Bronchodilators | SABA |
| d05465 | Ciclesonide | 2 | Respiratory Agents | Respiratory Inhalant Products | Inhaled Corticosteroids | ICS |
| d03276 | Dyphylline; Guaifenesin | 2 | Respiratory Agents | Antiasthmatic Combinations | - | Xanthine |
| d00758 | Aminophylline | 1 | Respiratory Agents | Bronchodilators | Methylxanthines | Xanthine |
| d04394 | Cromolyn Nasal | 1 | Topical Agents | Nasal Preparations | Nasal Antihistamines And Decongestants | MastCellStabilizer |
| d07868 | Azelastine; Fluticasone Nasal | 1 | Topical Agents | Nasal Preparations | Nasal Steroids | Antihistamine+ICS |
| d04881 | Omalizumab | 1 | Immunologic Agents | Immunosuppressive Agents | Other Immunosuppressants | mAb |
| d08675 | Benralizumab | 1 | Immunologic Agents | Immunosuppressive Agents | Interleukin Inhibitors | mAb |
| c00131 | Antiasthmatic Combinations - Unspecified | 1 | Respiratory Agents | Antiasthmatic Combinations | - | Antihistamine |
| d00754 | Bitolterol | 0 | Respiratory Agents | Bronchodilators | Adrenergic Bronchodilators | LABA |
| d00756 | Oxtriphylline | 0 | Respiratory Agents | Bronchodilators | Methylxanthines | Methylxanthines |
| d03273 | Guaifenesin; Theophylline | 0 | Respiratory Agents | Antiasthmatic Combinations | - | Methylxanthines |
| d03277 | Potassium Iodide; Theophylline | 0 | Respiratory Agents | Antiasthmatic Combinations | - | Methylxanthines |
| d03280 | Ephedrine; Phenobarbital; Theophylline | 0 | Respiratory Agents | Antiasthmatic Combinations | - | Methylxanthines |
| d03283 | Ephedrine; Hydroxyzine; Theophylline | 0 | Respiratory Agents | Antiasthmatic Combinations | - | Methylxanthines |
| d03287 | Ephedrine; Phenobarbital; Potassium Iodide; Theophylline | 0 | Respiratory Agents | Antiasthmatic Combinations | - | Methylxanthines |

Three-level therapeutic categories were obtained from the Multum Lexicon database.

Abbreviations: SABA, short-acting β2-agonists; LABA, long-acting β2-agonists; ICS, inhaled corticosteroids; SAMA, short-acting muscarinic antagonists; LAMA, long-acting muscarinic antagonists; LTRA, leukotriene receptor antagonists; mAb, monoclonal antibodies.

**eTable 2** **Asthma status of participants stratified by age**

| Characteristics | 1-11 years (N=28366, 14.9%) | 12-19 years (N=19653, 11.2%) | 20-39 years (N=22007, 27.5%) | 40-59 years (N=20644, 27.3%) | 60 years and over (N=23835, 19.2%) | *P* |
| --- | --- | --- | --- | --- | --- | --- |
| Lifetime asthma, n (%) |  |  |  |  |  | **<0.001** |
| No | 24598 (87.4) | 16017 (80.7) | 18521 (83.6) | 17787 (86.3) | 20866 (87.3) |  |
| Yes | 3768 (12.6) | 3636 (19.3) | 3486 (16.4) | 2857 (13.7) | 2969 (12.7) |  |
| Current asthma^a^, n (%) |  |  |  |  |  | **<0.001** |
| No | 95185 (91.2) | 23704 (91.7) | 15291 (88.8) | 18552 (91.5) | 17529 (91.4) |  |
| Yes | 9559 (8.8) | 2413 (8.3) | 1903 (11.2) | 1692 (8.5) | 1716 (8.6) |  |
| Asthma attack in past year among  individuals with current asthma^a^, n (%) |  |  |  |  |  | **<0.001** |
| No | 946 (38.6) | 1063 (54.3) | 884 (52.6) | 767 (45.3) | 1089 (59.1) |  |
| Yes | 1463 (61.4) | 839 (45.7) | 807 (47.4) | 947 (54.7) | 734 (40.9) |  |
| Asthma-related emergency visits  in past year among individuals with  current asthma^a^, n (%) |  |  |  |  |  | **<0.001** |
| No | 1279 (70.5) | 1123 (84.5) | 1060 (81.5) | 1153 (84.5) | 1169 (86.6) |  |
| Yes | 703 (29.5) | 266 (15.5) | 288 (18.5) | 289 (15.5) | 229 (13.4) |  |
| Asthma medication use  among asthma population^b^, n (%) |  |  |  |  |  | **<0.001** |
| No | 424 (27.0) | 528 (43.5) | 818 (61.9) | 1108 (60.5) | 1251 (56.3) |  |
| Yes | 1267 (73.0) | 820 (56.5) | 548 (38.1) | 731 (39.5) | 970 (43.7) |  |

All categorical variables were presented as frequency (weighted percentage). Difference was tested using the weighted Chi-square tests.

^a^Due to inconsistencies in the question routing logic for three asthma-related items (current asthma, asthma attack in past year, and asthma-related emergency visits in past year) between the 1999-2000 cycle and other cycles, data from the 1999-2000 cycle were not used for these three asthma status variables.

^b^Since NHANES 2021-2023 has not yet released detailed prescription medication data, the analysis of medication use among the asthma population is limited to prescription drug information from 1999-2020.

**eTable 3** **Asthma status of participants stratified by race**

| Characteristics | Mexican American (N=22670, 9.6%) | Other Hispanic (N=9918, 6.6%) | Non-Hispanic White (N=44061, 63.7%) | Non-Hispanic Black (N=26302, 12.0%) | Other Race (N=11554, 8.1%) | *P* |
| --- | --- | --- | --- | --- | --- | --- |
| Lifetime asthma, n (%) |  |  |  |  |  | **<0.001** |
| No | 20459 (90.1) | 8249 (83.7) | 37616 (85.3) | 21519 (82.2) | 9946 (85.7) |  |
| Yes | 2211 (9.9) | 1669 (16.3) | 6445 (14.7) | 4783 (17.8) | 1608 (14.3) |  |
| Current asthma^a^, n (%) |  |  |  |  |  | **<0.001** |
| No | 95185 (91.2) | 18354 (94.6) | 8428 (91.0) | 37018 (91.2) | 21140 (88.5) |  |
| Yes | 9559 (8.8) | 1103 (5.4) | 921 (9.0) | 3681 (8.8) | 2934 (11.5) |  |
| Asthma attack in past year among  individuals with current asthma^a^, n (%) |  |  |  |  |  | 0.061 |
| No | 552 (51.7) | 427 (48.3) | 1814 (50.0) | 1526 (52.7) | 430 (46.1) |  |
| Yes | 549 (48.3) | 493 (51.7) | 1858 (50.0) | 1403 (47.3) | 487 (53.9) |  |
| Asthma-related emergency visits  in past year among individuals with  current asthma^a^, n (%) |  |  |  |  |  | **<0.001** |
| No | 610 (78.2) | 540 (72.9) | 2425 (86.5) | 1544 (71.1) | 665 (79.7) |  |
| Yes | 213 (21.8) | 224 (27.1) | 452 (13.5) | 726 (28.9) | 160 (20.3) |  |
| Asthma medication use  among asthma population^b^, n (%) |  |  |  |  |  | **0.002** |
| No | 421 (47.2) | 372 (53.6) | 1934 (55.7) | 1049 (50.5) | 353 (54.6) |  |
| Yes | 576 (52.8) | 378 (46.4) | 1736 (44.3) | 1289 (49.5) | 357 (45.4) |  |

All categorical variables were presented as frequency (weighted percentage). Difference was tested using the weighted Chi-square tests.

^a^Due to inconsistencies in the question routing logic for three asthma-related items (current asthma, asthma attack in past year, and asthma-related emergency visits in past year) between the 1999-2000 cycle and other cycles, data from the 1999-2000 cycle were not used for these three asthma status variables.

^b^Since NHANES 2021-2023 has not yet released detailed prescription medication data, the analysis of medication use among the asthma population is limited to prescription drug information from 1999-2020.

**eTable 4** **Asthma status of participants stratified by education level**

| Characteristics | Less than high school (N=16801, 16.5%) | High school (N=15367, 24.4%) | Some college or AA degree (N=18920, 30.5%) | College graduate or above (N=15253, 28.6%) | *P* |
| --- | --- | --- | --- | --- | --- |
| Lifetime asthma, n (%) |  |  |  |  | **<0.001** |
| No | 14749 (87.0) | 13271 (86.0) | 15866 (83.9) | 13160 (86.2) |  |
| Yes | 2052 (13.0) | 2096 (14.0) | 3054 (16.1) | 2093 (13.8) |  |
| Current asthma^a^, n (%) |  |  |  |  | **<0.001** |
| No | 56080 (91.5) | 13630 (91.5) | 13004 (91.5) | 16094 (90.5) |  |
| Yes | 5231 (8.5) | 1232 (8.5) | 1219 (8.5) | 1701 (9.5) |  |
| Asthma attack in past year among  individuals with current asthma^a^, n (%) |  |  |  |  | **<0.001** |
| No | 678 (55.0) | 681 (55.8) | 814 (45.6) | 564 (53.2) |  |
| Yes | 547 (45.0) | 535 (44.2) | 886 (54.4) | 511 (46.8) |  |
| Asthma-related emergency visits  in past year among individuals with  current asthma^a^, n (%) |  |  |  |  | **<0.001** |
| No | 665 (75.9) | 774 (84.0) | 1144 (82.9) | 794 (90.3) |  |
| Yes | 243 (24.1) | 192 (16.0) | 261 (17.1) | 103 (9.7) |  |
| Asthma medication use  among asthma population^b^, n (%) |  |  |  |  | 0.089 |
| No | 752 (55.0) | 705 (59.2) | 1057 (61.4) | 659 (60.4) |  |
| Yes | 594 (45.0) | 501 (40.8) | 696 (38.6) | 454 (39.6) |  |

All categorical variables were presented as frequency (weighted percentage). Difference was tested using the weighted Chi-square tests.

^a^Due to inconsistencies in the question routing logic for three asthma-related items (current asthma, asthma attack in past year, and asthma-related emergency visits in past year) between the 1999-2000 cycle and other cycles, data from the 1999-2000 cycle were not used for these three asthma status variables.

^b^Since NHANES 2021-2023 has not yet released detailed prescription medication data, the analysis of medication use among the asthma population is limited to prescription drug information from 1999-2020.

**eTable 5** **Asthma status of participants stratified by PIR**

| Characteristics | PIR <1.3 (N=36910, 24.1%) | PIR 1.3–3.5 (N=37914, 36.3%) | PIR >3.5 (N=27916, 39.6%) | *P* |
| --- | --- | --- | --- | --- |
| Lifetime asthma, n (%) |  |  |  | **<0.001** |
| No | 30864 (82.7) | 32604 (85.6) | 24058 (86.2) |  |
| Yes | 6046 (17.3) | 5310 (14.4) | 3858 (13.8) |  |
| Current asthma^a^, n (%) |  |  |  | **<0.001** |
| No | 29950 (88.8) | 31943 (91.4) | 23826 (92.3) |  |
| Yes | 3712 (11.2) | 2985 (8.6) | 2017 (7.7) |  |
| Asthma attack in past year among  individuals with current asthma^a^, n (%) |  |  |  | **0.022** |
| No | 1797 (47.4) | 1456 (48.6) | 1041 (52.1) |  |
| Yes | 1906 (52.6) | 1526 (51.4) | 973 (47.9) |  |
| Asthma-related emergency visits  in past year among individuals with  current asthma^a^, n (%) |  |  |  | **<0.001** |
| No | 2087 (75.7) | 1824 (81.4) | 1334 (88.6) |  |
| Yes | 855 (24.3) | 530 (18.6) | 234 (11.4) |  |
| Asthma medication use  among asthma population^b^, n (%) |  |  |  | **0.003** |
| No | 1376 (49.8) | 1353 (54.5) | 1055 (56.9) |  |
| Yes | 1679 (50.2) | 1379 (45.5) | 962 (43.1) |  |

All categorical variables were presented as frequency (weighted percentage). Difference was tested using the weighted Chi-square tests.

^a^Due to inconsistencies in the question routing logic for three asthma-related items (current asthma, asthma attack in past year, and asthma-related emergency visits in past year) between the 1999-2000 cycle and other cycles, data from the 1999-2000 cycle were not used for these three asthma status variables.

Abbreviations: PIR, poverty-to-income ratio.

^b^Since NHANES 2021-2023 has not yet released detailed prescription medication data, the analysis of medication use among the asthma population is limited to prescription drug information from 1999-2020.

**eTable 6 Prevalence of different asthma statuses across cycles in the general population and age-****stratified subgroups**

| Cycle | Lifetime asthma  (N=114505) | Current asthma^a^  (N=104744) | Current asthma | |
| --- | --- | --- | --- | --- |
|  |  |  | Asthma attack  in past year^a^  (N=9539) | Asthma-related emergency  visits in past year^a^  (N=7559) |
| **1-11 years** |  |  |  |  |
| Total (N=28366) | 12.6 (12.0, 13.3) | 8.3 (7.9, 8.8) | 61.4 (58.7, 64.1) | 29.5 (27.3, 31.9) |
| 1999-2000 (N=2194) | 10.6 (14.3, 19.1) | - | - | - |
| 2001-2002 (N=2569) | 10.7 (12.8, 15.3) | 6.9 (8.5, 10.3) | 56.9 (66.0, 74.0) | 28.0 (35.1, 43.0) |
| 2003-2004 (N=2293) | 10.1 (11.9, 13.9) | 6.1 (7.5, 9.2) | 59.5 (68.2, 75.9) | 27.9 (39.5, 52.4) |
| 2005-2006 (N=2552) | 11.4 (13.4, 15.6) | 8.2 (9.7, 11.4) | 62.5 (74.5, 83.7) | 25.0 (32.2, 40.3) |
| 2007-2008 (N=2493) | 12.4 (14.0, 15.8) | 7.5 (8.7, 10.0) | 54.0 (61.7, 69.0) | 26.5 (35.2, 44.9) |
| 2009-2010 (N=2542) | 11.2 (12.9, 14.7) | 7.0 (8.3, 9.9) | 45.2 (54.6, 63.7) | 32.0 (41.0, 50.7) |
| 2011-2012 (N=2526) | 11.4 (13.9, 16.7) | 7.4 (9.1, 11.2) | 53.8 (61.8, 69.2) | 18.9 (26.0, 34.5) |
| 2013-2014 (N=2565) | 11.0 (13.3, 15.9) | 7.5 (8.6, 9.9) | 49.4 (56.5, 63.3) | 20.4 (27.4, 35.7) |
| 2015-2016 (N=2535) | 10.9 (12.4, 14.0) | 7.2 (8.6, 10.3) | 49.5 (60.5, 70.5) | 21.8 (26.4, 31.6) |
| 2017-2020 (N=3746) | 10.1 (11.9, 13.9) | 6.6 (8.1, 9.8) | 48.4 (55.1, 61.6) | 19.2 (25.1, 32.0) |
| 2021-2023 (N=2351) | 6.3 (8.3, 10.8) | 4.8 (6.4, 8.4) | 47.1 (56.6, 65.6) | 16.5 (21.6, 27.7) |
| Average change per cycle^b^ | -0.30 (-0.60, 0.01) | -0.12 (-0.34, 0.10) | **-1.55 (-2.74, -0.36)** | **-1.76 (-2.81, -0.71)** |
| *P*-value for trend^b^ | 0.054 | 0.248 | **0.017** | **0.005** |
|  |  |  |  |  |
| **12-19 years** |  |  |  |  |
| Total (N=19653) | 19.3 (18.5, 20.1) | 11.2 (10.6, 11.8) | 45.7 (42.2, 49.1) | 15.5 (13.2, 18.1) |
| 1999-2000 (N=2415) | 14.5 (17.1, 20.0) | - | - | - |
| 2001-2002 (N=2484) | 14.1 (16.1, 18.3) | 7.9 (9.4, 11.2) | 40.7 (54.2, 67.1) | 15.7 (26.0, 39.8) |
| 2003-2004 (N=2299) | 16.2 (18.4, 20.8) | 8.6 (10.1, 11.7) | 46.0 (52.5, 58.9) | 18.2 (27.3, 38.8) |
| 2005-2006 (N=2285) | 16.6 (18.7, 21.0) | 9.3 (10.8, 12.6) | 41.3 (54.8, 67.6) | 12.5 (21.1, 33.5) |
| 2007-2008 (N=1237) | 17.2 (20.4, 23.9) | 9.8 (11.6, 13.7) | 40.3 (50.8, 61.2) | 9.9 (19.8, 35.6) |
| 2009-2010 (N=1339) | 15.2 (17.7, 20.6) | 8.8 (10.3, 12.1) | 32.2 (43.4, 55.3) | 11.1 (22.7, 40.9) |
| 2011-2012 (N=1272) | 20.1 (22.6, 25.3) | 11.5 (13.4, 15.6) | 33.6 (49.8, 66.1) | 9.9 (18.1, 30.6) |
| 2013-2014 (N=1431) | 17.0 (20.3, 24.1) | 9.5 (11.5, 13.7) | 32.8 (41.0, 49.9) | 6.5 (10.4, 16.3) |
| 2015-2016 (N=1314) | 17.2 (20.5, 24.3) | 9.4 (11.5, 14.1) | 29.2 (39.7, 51.3) | 10.5 (16.7, 25.6) |
| 2017-2020 (N=2000) | 17.4 (19.7, 22.2) | 8.9 (11.0, 13.4) | 23.4 (33.2, 44.8) | 6.5 (10.0, 14.9) |
| 2021-2023 (N=1577) | 17.7 (20.3, 23.3) | 10.3 (12.1, 14.1) | 38.0 (45.9, 54.1) | 4.0 (7.8, 14.8) |
| Average change per cycle^b^ | **0.38 (0.08, 0.69)** | 0.22 (-0.02, 0.46) | **-1.86 (-3.03, -0.70)** | **-2.06 (-2.78, -1.35)** |
| *P*-value for trend^b^ | **0.020** | 0.071 | **0.006** | **<0.001** |
|  |  |  |  |  |
| **20-39 years** |  |  |  |  |
| Total (N=22007) | 16.4 (15.8, 17.1) | 8.5 (8.0, 9.0) | 47.4 (44.4, 50.4) | 18.5 (16.1, 21.1) |
| 1999-2000 (N=1695) | 10.0 (12.3, 15.2) | - | - | - |
| 2001-2002 (N=1924) | 10.4 (12.9, 15.8) | 5.6 (7.6, 10.1) | 49.5 (57.2, 64.5) | 24.3 (33.3, 43.7) |
| 2003-2004 (N=1739) | 11.8 (13.7, 15.9) | 6.2 (7.6, 9.3) | 37.5 (46.2, 55.2) | 9.3 (17.8, 31.3) |
| 2005-2006 (N=1922) | 14.1 (16.1, 18.3) | 6.1 (7.7, 9.5) | 44.8 (52.5, 60.1) | 26.3 (37.5, 50.2) |
| 2007-2008 (N=1908) | 13.3 (15.6, 18.2) | 5.5 (6.7, 8.2) | 41.1 (48.2, 55.3) | 17.0 (28.8, 44.4) |
| 2009-2010 (N=2080) | 13.6 (15.2, 17.0) | 5.7 (7.0, 8.6) | 40.7 (50.1, 59.4) | 15.3 (26.1, 40.8) |
| 2011-2012 (N=1957) | 13.7 (17.0, 20.8) | 7.0 (8.9, 11.3) | 46.1 (55.4, 64.2) | 9.9 (16.9, 27.5) |
| 2013-2014 (N=1952) | 17.0 (18.7, 20.5) | 7.5 (8.9, 10.4) | 37.0 (47.1, 57.4) | 10.6 (14.3, 19.1) |
| 2015-2016 (N=1952) | 16.6 (18.3, 20.0) | 8.2 (9.4, 10.9) | 37.0 (47.5, 58.1) | 7.5 (12.5, 20.0) |
| 2017-2020 (N=2796) | 15.8 (18.0, 20.4) | 7.4 (9.2, 11.4) | 29.0 (38.0, 47.9) | 11.2 (16.8, 24.5) |
| 2021-2023 (N=2082) | 18.5 (21.2, 24.1) | 9.5 (11.0, 12.8) | 33.5 (43.2, 53.4) | 8.1 (13.4, 21.3) |
| Average change per cycle^b^ | **0.77 (0.58, 0.96)** | **0.36 (0.16, 0.56)** | **-1.25 (-2.38, -0.12)** | **-2.20 (-3.81, -0.59)** |
| *P*-value for trend^b^ | **<0.001** | **0.003** | **0.034** | **0.014** |
|  |  |  |  |  |
| **40-59 years** |  |  |  |  |
| Total (N=20644) | 13.7 (13.0, 14.3) | 8.6 (8.1, 9.3) | 54.7 (51.6, 57.7) | 15.5 (13.3, 18.0) |
| 1999-2000 (N=1349) | 9.8 (11.3, 13.1) | - | - | - |
| 2001-2002 (N=1612) | 9.3 (11.5, 14.1) | 5.9 (7.4, 9.3) | 45.5 (57.3, 68.4) | 9.0 (17.5, 31.1) |
| 2003-2004 (N=1396) | 10.4 (12.6, 15.3) | 6.3 (7.9, 9.9) | 36.3 (48.3, 60.5) | 9.8 (20.0, 36.5) |
| 2005-2006 (N=1484) | 11.5 (13.9, 16.6) | 7.5 (9.2, 11.3) | 56.1 (66.8, 76.1) | 10.6 (20.1, 34.9) |
| 2007-2008 (N=1869) | 11.4 (13.8, 16.7) | 6.2 (8.8, 12.2) | 51.2 (60.2, 68.6) | 14.5 (21.8, 31.4) |
| 2009-2010 (N=2059) | 10.3 (11.9, 13.7) | 5.1 (6.3, 7.8) | 39.7 (52.6, 65.2) | 18.1 (31.3, 48.5) |
| 2011-2012 (N=1811) | 11.1 (13.8, 17.1) | 6.3 (8.2, 10.8) | 45.5 (53.0, 60.3) | 4.9 (9.2, 16.6) |
| 2013-2014 (N=1972) | 11.6 (13.5, 15.5) | 7.4 (9.0, 10.9) | 35.6 (48.2, 61.1) | 9.3 (14.2, 21.1) |
| 2015-2016 (N=1865) | 11.6 (13.7, 16.2) | 6.4 (8.2, 10.4) | 40.6 (53.4, 65.7) | 7.9 (12.9, 20.4) |
| 2017-2020 (N=3009) | 13.0 (15.1, 17.4) | 7.6 (9.1, 10.9) | 43.8 (51.1, 58.3) | 10.5 (15.3, 21.8) |
| 2021-2023 (N=2218) | 15.5 (17.9, 20.6) | 9.9 (12.0, 14.5) | 46.9 (56.1, 64.8) | 6.5 (11.5, 19.3) |
| Average change per cycle^b^ | **0.45 (0.20, 0.70)** | 0.29 (-0.04, 0.61) | -0.57 (-2.03, 0.89) | -1.02 (-2.52, 0.48) |
| *P*-value for trend^b^ | **0.003** | 0.077 | 0.392 | 0.157 |
|  |  |  |  |  |
| **60 years and over** |  |  |  |  |
| Total (N=23835) | 12.7 (12.0, 13.4) | 8.3 (7.6, 8.9) | 40.9 (37.9, 44.0) | 13.4 (11.4, 15.6) |
| 1999-2000 (N=1831) | 9.4 (11.5, 13.9) | - | - | - |
| 2001-2002 (N=1871) | 6.9 (8.6, 10.5) | 4.1 (5.6, 7.5) | 23.3 (35.4, 49.8) | 20.3 (44.3, 71.2) |
| 2003-2004 (N=1899) | 8.7 (10.8, 13.4) | 5.9 (7.5, 9.5) | 25.9 (34.3, 43.8) | 6.8 (10.6, 16.2) |
| 2005-2006 (N=1566) | 9.3 (11.1, 13.2) | 5.7 (7.3, 9.3) | 35.8 (42.7, 50.0) | 11.5 (24.5, 44.8) |
| 2007-2008 (N=2152) | 9.6 (11.0, 12.7) | 6.4 (7.6, 9.0) | 24.2 (34.7, 47.0) | 17.4 (26.2, 37.6) |
| 2009-2010 (N=2072) | 10.8 (13.0, 15.7) | 6.9 (8.5, 10.4) | 18.7 (29.4, 43.1) | 19.3 (32.2, 48.6) |
| 2011-2012 (N=1788) | 10.9 (13.5, 16.7) | 5.7 (8.3, 11.9) | 30.7 (42.2, 54.7) | 7.9 (14.1, 23.7) |
| 2013-2014 (N=1840) | 12.0 (14.7, 18.0) | 8.2 (10.3, 12.8) | 35.7 (46.0, 56.6) | 7.1 (12.1, 20.0) |
| 2015-2016 (N=1898) | 12.4 (15.2, 18.5) | 7.3 (9.7, 12.9) | 36.9 (43.6, 50.5) | 5.6 (10.2, 17.8) |
| 2017-2020 (N=3418) | 10.6 (12.5, 14.7) | 5.6 (7.2, 9.2) | 34.0 (44.0, 54.5) | 7.0 (9.9, 13.9) |
| 2021-2023 (N=3500) | 13.3 (14.7, 16.1) | 8.5 (9.7, 10.9) | 37.3 (44.3, 51.4) | 7.3 (9.6, 12.6) |
| Average change per cycle^b^ | **0.50 (0.22, 0.77)** | **0.33 (0.06, 0.61)** | **1.20 (0.04, 2.37)** | **-2.72 (-5.05, -0.39)** |
| *P*-value for trend^b^ | **0.003** | **0.024** | **0.044** | **0.027** |

All prevalences and 95% CI were weighted.

^a^Due to inconsistencies in the question routing logic for three asthma-related items (current asthma, asthma attack in past year, and asthma-related emergency visits in past year) between the 1999-2000 cycle and other cycles, data from the 1999-2000 cycle were not used for these three asthma status variables.

^b^The estimate (β), 95% CI, and *P*-value for trend were obtained from linear regression models that included prevalence per cycle as the dependent variable and NHANES cycle as the continuous independent variable. The estimate (β) represents the average percentage change in prevalence per cycle.

Bold indicates significant estimates, showing statistically significant increasing or decreasing temporal trends in prevalence.

**eTable 7 Prevalence of different asthma statuses across cycles in the general population and race-stratified subgroups**

| Cycle | Lifetime asthma  (N=114505) | Current asthma^a^  (N=104744) | Current asthma | |
| --- | --- | --- | --- | --- |
|  |  |  | Asthma attack  in past year^a^  (N=9539) | Asthma-related emergency  visits in past year^a^  (N=7559) |
| **Mexican American** |  |  |  |  |
| Total (N=22670) | 9.9 (9.3, 10.6) | 5.4 (5.0, 5.9) | 48.3 (44.7, 52.0) | 21.8 (18.4, 25.6) |
| 1999-2000 (N=3174) | 6.1 (7.5, 9.2) | - | - | - |
| 2001-2002 (N=2592) | 5.9 (7.6, 9.8) | 2.8 (3.9, 5.4) | 48.4 (68.1, 82.9) | 21.0 (37.6, 57.8) |
| 2003-2004 (N=2349) | 6.7 (8.3, 10.2) | 4.2 (5.2, 6.5) | 38.5 (50.2, 61.8) | 25.2 (37.2, 51.1) |
| 2005-2006 (N=2612) | 6.4 (8.5, 11.1) | 3.5 (4.9, 6.9) | 41.1 (50.3, 59.5) | 22.5 (32.9, 45.3) |
| 2007-2008 (N=1988) | 6.6 (8.1, 9.9) | 3.0 (3.6, 4.3) | 38.4 (52.3, 65.9) | 11.2 (27.1, 52.4) |
| 2009-2010 (N=2237) | 7.0 (8.4, 10.0) | 3.4 (4.7, 6.3) | 36.8 (51.8, 66.5) | 20.3 (34.2, 51.4) |
| 2011-2012 (N=1261) | 7.4 (10.0, 13.5) | 3.8 (5.2, 7.1) | 22.2 (43.6, 67.6) | 10.9 (28.3, 56.2) |
| 2013-2014 (N=1645) | 10.5 (12.6, 15.1) | 5.9 (7.0, 8.3) | 34.6 (40.5, 46.7) | 9.1 (14.6, 22.6) |
| 2015-2016 (N=1823) | 8.4 (10.2, 12.3) | 3.8 (5.4, 7.5) | 24.1 (34.5, 46.6) | 9.8 (16.9, 27.6) |
| 2017-2020 (N=1899) | 10.2 (11.8, 13.7) | 5.5 (6.7, 8.2) | 38.5 (47.3, 56.2) | 12.1 (20.2, 31.8) |
| 2021-2023 (N=1090) | 10.1 (13.2, 17.2) | 4.5 (6.3, 8.9) | 46.8 (60.7, 73.0) | 6.4 (11.4, 19.5) |
| Average change per cycle^b^ | **0.56 (0.35, 0.77)** | **0.28 (0.07, 0.48)** | -1.27 (-3.64, 1.10) | **-2.90 (-3.99, -1.81)** |
| *P*-value for trend^b^ | **<0.001** | **0.014** | 0.250 | **<0.001** |
|  |  |  |  |  |
| **Other Hispanic** |  |  |  |  |
| Total (N=9918) | 16.3 (15.2, 17.5) | 9.0 (8.2, 9.8) | 51.7 (47.4, 55.9) | 27.1 (22.6, 32.1) |
| 1999-2000 (N=551) | 10.6 (13.8, 17.7) | - | - | - |
| 2001-2002 (N=487) | 13.8 (18.0, 23.2) | 6.7 (10.7, 16.6) | 29.1 (49.8, 70.5) | 0.0 (57.3, NaN) |
| 2003-2004 (N=316) | 7.7 (13.1, 21.4) | 3.9 (6.6, 10.9) | 27.8 (54.6, 79.0) | 7.3 (43.9, 88.5) |
| 2005-2006 (N=329) | 8.9 (14.5, 22.8) | 4.9 (7.7, 12.0) | 28.6 (56.8, 81.2) | 3.8 (50.4, 96.3) |
| 2007-2008 (N=1151) | 13.1 (17.0, 21.8) | 7.1 (9.5, 12.6) | 41.7 (48.2, 54.8) | 40.0 (58.7, 75.3) |
| 2009-2010 (N=1074) | 10.8 (13.3, 16.2) | 6.6 (8.2, 10.1) | 40.9 (50.5, 60.1) | 22.0 (36.9, 54.7) |
| 2011-2012 (N=1030) | 12.2 (15.7, 19.9) | 6.9 (9.0, 11.7) | 47.7 (59.4, 70.1) | 16.3 (28.1, 44.0) |
| 2013-2014 (N=915) | 11.6 (14.7, 18.5) | 6.0 (8.0, 10.5) | 40.1 (49.7, 59.2) | 16.1 (23.6, 33.2) |
| 2015-2016 (N=1254) | 15.7 (17.7, 20.0) | 7.0 (8.5, 10.3) | 42.8 (58.2, 72.1) | 19.1 (28.3, 39.8) |
| 2017-2020 (N=1466) | 15.1 (17.9, 21.1) | 7.6 (9.2, 11.1) | 32.5 (42.5, 53.0) | 9.4 (17.5, 30.1) |
| 2021-2023 (N=1345) | 14.3 (18.1, 22.5) | 7.5 (9.8, 12.7) | 40.9 (56.3, 70.5) | 8.4 (15.1, 25.8) |
| Average change per cycle^b^ | 0.31 (-0.08, 0.70) | 0.06 (-0.25, 0.38) | -0.04 (-1.47, 1.40) | **-4.78 (-6.59, -2.96)** |
| *P*-value for trend^b^ | 0.108 | 0.647 | 0.953 | **<0.001** |
|  |  |  |  |  |
| **Non-Hispanic White** |  |  |  |  |
| Total (N=44061) | 14.7 (14.3, 15.2) | 8.8 (8.4, 9.2) | 50.0 (48.0, 52.0) | 13.5 (12.1, 15.2) |
| 1999-2000 (N=3242) | 10.7 (13.0, 15.6) | - | - | - |
| 2001-2002 (N=4399) | 10.2 (11.9, 13.9) | 6.5 (7.7, 9.0) | 50.7 (56.8, 62.8) | 17.0 (22.0, 28.1) |
| 2003-2004 (N=3965) | 12.3 (13.2, 14.2) | 7.1 (8.0, 9.0) | 44.0 (49.7, 55.4) | 11.4 (18.1, 27.5) |
| 2005-2006 (N=3770) | 13.9 (15.1, 16.4) | 8.2 (9.1, 10.1) | 58.2 (63.4, 68.4) | 16.5 (22.6, 30.2) |
| 2007-2008 (N=3938) | 13.4 (14.9, 16.6) | 6.9 (8.4, 10.1) | 45.1 (51.6, 58.0) | 14.4 (20.5, 28.2) |
| 2009-2010 (N=4275) | 12.5 (13.7, 15.0) | 6.4 (7.3, 8.3) | 36.5 (42.7, 49.1) | 15.2 (23.4, 34.2) |
| 2011-2012 (N=2857) | 13.8 (16.2, 18.8) | 7.6 (9.5, 11.7) | 47.0 (53.3, 59.4) | 6.7 (11.8, 19.8) |
| 2013-2014 (N=3524) | 14.8 (16.1, 17.6) | 8.6 (9.6, 10.8) | 40.8 (46.2, 51.7) | 7.7 (11.3, 16.1) |
| 2015-2016 (N=2932) | 15.2 (16.2, 17.3) | 8.7 (9.6, 10.7) | 41.7 (48.7, 55.8) | 6.8 (9.5, 13.1) |
| 2017-2020 (N=5032) | 13.5 (15.0, 16.7) | 7.1 (8.5, 10.0) | 36.0 (43.2, 50.7) | 7.6 (10.3, 13.9) |
| 2021-2023 (N=6127) | 15.5 (16.7, 18.1) | 9.4 (10.4, 11.5) | 43.7 (47.7, 51.7) | 5.5 (8.7, 13.4) |
| Average change per cycle^b^ | **0.40 (0.20, 0.60)** | **0.22 (0.02, 0.42)** | -1.26 (-2.62, 0.11) | **-1.69 (-2.55, -0.84)** |
| *P*-value for trend^b^ | **0.001** | **0.037** | 0.066 | **0.002** |
|  |  |  |  |  |
| **Non-Hispanic Black** |  |  |  |  |
| Total (N=26302) | 17.8 (17.2, 18.5) | 11.5 (11.0, 12.1) | 47.3 (44.9, 49.6) | 28.9 (26.6, 31.4) |
| 1999-2000 (N=2152) | 14.0 (15.3, 16.8) | - | - | - |
| 2001-2002 (N=2566) | 11.9 (14.1, 16.6) | 7.1 (8.5, 10.0) | 41.5 (50.4, 59.3) | 36.1 (49.6, 63.2) |
| 2003-2004 (N=2560) | 14.9 (16.6, 18.6) | 8.9 (10.5, 12.3) | 41.8 (50.4, 59.0) | 32.0 (40.5, 49.5) |
| 2005-2006 (N=2621) | 14.5 (16.4, 18.6) | 9.3 (10.5, 11.8) | 37.0 (44.1, 51.5) | 39.8 (47.8, 55.9) |
| 2007-2008 (N=2143) | 14.8 (17.2, 19.8) | 8.8 (11.0, 13.8) | 51.3 (56.9, 62.3) | 25.0 (38.1, 53.1) |
| 2009-2010 (N=1890) | 16.6 (19.7, 23.1) | 10.0 (12.6, 15.7) | 41.5 (52.3, 62.9) | 36.5 (45.4, 54.6) |
| 2011-2012 (N=2587) | 16.7 (18.9, 21.4) | 10.0 (11.9, 14.1) | 41.6 (48.0, 54.5) | 18.0 (23.9, 31.0) |
| 2013-2014 (N=2183) | 16.0 (18.6, 21.6) | 10.6 (12.8, 15.3) | 42.5 (48.2, 53.9) | 22.6 (27.6, 33.1) |
| 2015-2016 (N=2054) | 16.6 (19.1, 21.9) | 10.1 (12.0, 14.3) | 37.0 (44.4, 52.2) | 18.3 (22.7, 27.7) |
| 2017-2020 (N=3971) | 17.3 (19.1, 21.0) | 10.6 (11.8, 13.0) | 35.8 (40.9, 46.3) | 19.9 (24.4, 29.5) |
| 2021-2023 (N=1575) | 17.0 (19.4, 22.0) | 11.1 (13.2, 15.7) | 34.0 (43.5, 53.5) | 12.4 (18.5, 26.8) |
| Average change per cycle^b^ | **0.49 (0.28, 0.70)** | **0.39 (0.18, 0.60)** | -0.95 (-1.98, 0.08) | **-3.46 (-4.85, -2.08)** |
| *P*-value for trend^b^ | **<0.001** | **0.002** | 0.065 | **<0.001** |
|  |  |  |  |  |
| **Other Race** |  |  |  |  |
| Total (N=11554) | 14.3 (13.3, 15.3) | 8.3 (7.5, 9.1) | 53.9 (49.6, 58.3) | 20.3 (16.5, 24.8) |
| 1999-2000 (N=365) | 8.0 (12.6, 19.1) | - | - | - |
| 2001-2002 (N=416) | 8.8 (11.5, 14.9) | 4.6 (6.6, 9.4) | 27.4 (60.0, 85.6) | 4.1 (22.5, 66.2) |
| 2003-2004 (N=436) | 9.7 (13.4, 18.3) | 4.5 (6.8, 10.1) | 21.0 (33.8, 49.4) | 2.8 (9.6, 28.0) |
| 2005-2006 (N=477) | 9.2 (13.7, 19.8) | 4.4 (6.9, 10.9) | 30.9 (61.1, 84.7) | 6.3 (27.6, 68.2) |
| 2007-2008 (N=439) | 10.2 (14.4, 19.9) | 5.6 (8.6, 13.0) | 32.2 (51.2, 69.8) | 3.9 (18.6, 56.2) |
| 2009-2010 (N=616) | 10.6 (13.3, 16.5) | 4.6 (6.7, 9.7) | 27.2 (52.4, 76.4) | 11.6 (34.1, 67.1) |
| 2011-2012 (N=1619) | 9.9 (12.6, 16.0) | 5.4 (7.0, 9.0) | 41.9 (57.1, 71.0) | 9.2 (16.0, 26.6) |
| 2013-2014 (N=1493) | 12.5 (14.8, 17.4) | 6.0 (7.4, 9.2) | 51.3 (66.4, 78.8) | 7.8 (14.4, 25.0) |
| 2015-2016 (N=1501) | 10.6 (13.7, 17.4) | 5.8 (8.2, 11.6) | 46.9 (59.4, 70.9) | 10.6 (24.6, 47.3) |
| 2017-2020 (N=2601) | 11.7 (14.3, 17.3) | 6.4 (8.6, 11.4) | 44.9 (54.2, 63.1) | 14.8 (23.5, 35.1) |
| 2021-2023 (N=1591) | 14.4 (17.7, 21.5) | 8.6 (11.4, 15.0) | 38.5 (48.0, 57.6) | 11.1 (16.6, 24.1) |
| Average change per cycle^b^ | **0.34 (0.09, 0.60)** | **0.36 (0.09, 0.63)** | 0.47 (-1.94, 2.87) | -0.01 (-1.93, 1.92) |
| *P*-value for trend^b^ | **0.013** | **0.016** | 0.666 | 0.994 |

All prevalences and 95% CI were weighted.

^a^Due to inconsistencies in the question routing logic for three asthma-related items (current asthma, asthma attack in past year, and asthma-related emergency visits in past year) between the 1999-2000 cycle and other cycles, data from the 1999-2000 cycle were not used for these three asthma status variables.

^b^The estimate (β), 95% CI, and *P*-value for trend were obtained from linear regression models that included prevalence per cycle as the dependent variable and NHANES cycle as the continuous independent variable. The estimate (β) represents the average percentage change in prevalence per cycle.

Bold indicates significant estimates, showing statistically significant increasing or decreasing temporal trends in prevalence.

**eTable 8 Prevalence of different asthma statuses across cycles in the general population and education level-stratified subgroups**

| Cycle | Lifetime asthma  (N=114505) | Current asthma^a^  (N=104744) | Current asthma | |
| --- | --- | --- | --- | --- |
|  |  |  | Asthma attack  in past year^a^  (N=9539) | Asthma-related emergency  visits in past year^a^  (N=7559) |
| **Less than high school** |  |  |  |  |
| Total (N=16801) | 13.0 (12.2, 14.0) | 8.4 (7.7, 9.2) | 45.2 (41.3, 49.1) | 24.1 (21.1, 27.5) |
| 1999-2000 (N=1892) | 9.3 (11.9, 15.2) | - | - | - |
| 2001-2002 (N=1667) | 7.9 (9.5, 11.5) | 4.4 (6.0, 8.1) | 48.3 (59.4, 69.7) | 15.2 (31.9, 54.9) |
| 2003-2004 (N=1483) | 8.9 (11.9, 15.8) | 5.5 (8.3, 12.4) | 20.6 (29.7, 40.8) | 22.4 (35.9, 52.0) |
| 2005-2006 (N=1392) | 8.0 (11.1, 15.2) | 4.8 (6.8, 9.6) | 35.1 (51.5, 67.5) | 24.4 (36.9, 51.5) |
| 2007-2008 (N=1859) | 10.8 (13.9, 17.7) | 6.8 (8.7, 11.1) | 37.2 (49.8, 62.4) | 20.0 (29.2, 40.4) |
| 2009-2010 (N=1776) | 10.4 (12.9, 16.1) | 6.5 (8.4, 10.7) | 30.7 (44.1, 58.4) | 45.3 (59.6, 72.5) |
| 2011-2012 (N=1331) | 11.3 (14.5, 18.4) | 6.2 (8.1, 10.5) | 31.5 (38.3, 45.7) | 10.3 (14.3, 19.4) |
| 2013-2014 (N=1244) | 11.0 (13.9, 17.5) | 7.0 (8.8, 10.9) | 38.5 (49.6, 60.8) | 12.2 (18.0, 25.9) |
| 2015-2016 (N=1363) | 11.9 (14.3, 17.1) | 7.7 (9.3, 11.2) | 30.1 (44.5, 59.8) | 11.3 (16.1, 22.3) |
| 2017-2020 (N=1757) | 10.7 (13.7, 17.3) | 6.6 (9.1, 12.4) | 27.1 (39.0, 52.4) | 13.9 (21.4, 31.4) |
| 2021-2023 (N=1037) | 13.5 (17.9, 23.2) | 8.4 (11.7, 16.1) | 41.6 (51.5, 61.2) | 10.6 (17.9, 28.7) |
| Average change per cycle^b^ | **0.54 (0.27, 0.82)** | **0.42 (0.20, 0.65)** | -0.29 (-2.56, 1.98) | -2.49 (-5.63, 0.65) |
| *P*-value for trend^b^ | **0.002** | **0.002** | 0.775 | 0.105 |
|  |  |  |  |  |
| **High school** |  |  |  |  |
| Total (N=15367) | 14.0 (13.3, 14.8) | 8.5 (7.9, 9.2) | 43.2 (39.7, 46.7) | 16.1 (13.6, 19.0) |
| 1999-2000 (N=1097) | 8.8 (11.4, 14.8) | - | - | - |
| 2001-2002 (N=1264) | 10.0 (12.2, 14.9) | 6.2 (7.9, 10.0) | 29.6 (40.0, 51.5) | 27.1 (43.1, 60.8) |
| 2003-2004 (N=1268) | 9.1 (10.3, 11.6) | 4.9 (5.7, 6.6) | 37.9 (50.8, 63.5) | 2.5 (9.6, 30.8) |
| 2005-2006 (N=1179) | 10.0 (12.8, 16.2) | 5.2 (7.4, 10.4) | 40.3 (53.4, 66.0) | 21.2 (38.2, 58.7) |
| 2007-2008 (N=1460) | 11.3 (13.5, 15.9) | 5.6 (7.6, 10.3) | 31.5 (43.7, 56.7) | 5.9 (17.8, 42.6) |
| 2009-2010 (N=1423) | 12.4 (14.6, 17.2) | 5.7 (7.5, 9.8) | 32.1 (43.1, 54.9) | 18.8 (27.2, 37.6) |
| 2011-2012 (N=1168) | 9.7 (13.4, 18.2) | 5.6 (8.5, 12.7) | 28.7 (39.0, 50.4) | 8.0 (13.9, 22.9) |
| 2013-2014 (N=1303) | 12.1 (15.0, 18.5) | 7.3 (9.9, 13.1) | 24.4 (38.3, 54.3) | 8.6 (15.6, 26.7) |
| 2015-2016 (N=1235) | 12.7 (15.7, 19.3) | 6.5 (8.2, 10.3) | 38.1 (51.7, 65.0) | 8.4 (14.4, 23.6) |
| 2017-2020 (N=2222) | 13.2 (15.2, 17.5) | 8.0 (9.4, 11.1) | 25.9 (32.9, 40.7) | 6.9 (11.1, 17.4) |
| 2021-2023 (N=1748) | 16.0 (18.1, 20.3) | 9.8 (11.7, 13.9) | 40.3 (50.3, 60.4) | 7.0 (11.8, 19.4) |
| Average change per cycle^b^ | **0.60 (0.39, 0.81)** | **0.44 (0.18, 0.70)** | -0.37 (-2.21, 1.47) | **-2.49 (-4.95, -0.02)** |
| *P*-value for trend^b^ | **<0.001** | **0.005** | 0.654 | **0.049** |
|  |  |  |  |  |
| **Some college or AA degree** |  |  |  |  |
| Total (N=18920) | 16.1 (15.5, 16.8) | 9.4 (8.9, 10.0) | 55.1 (52.3, 57.8) | 17.3 (14.9, 20.0) |
| 1999-2000 (N=1070) | 9.1 (11.4, 14.1) | - | - | - |
| 2001-2002 (N=1377) | 9.3 (11.6, 14.4) | 5.5 (7.2, 9.5) | 58.2 (66.9, 74.5) | 9.4 (18.0, 31.9) |
| 2003-2004 (N=1356) | 13.0 (15.0, 17.2) | 7.3 (8.8, 10.5) | 33.6 (45.4, 57.8) | 9.5 (17.9, 31.1) |
| 2005-2006 (N=1414) | 14.9 (17.9, 21.4) | 7.8 (9.9, 12.6) | 52.8 (59.5, 65.9) | 9.8 (21.4, 40.6) |
| 2007-2008 (N=1505) | 12.7 (15.4, 18.6) | 7.0 (8.9, 11.3) | 47.2 (55.4, 63.3) | 17.7 (32.4, 51.5) |
| 2009-2010 (N=1740) | 12.9 (14.6, 16.6) | 6.5 (7.6, 9.0) | 37.9 (46.9, 56.2) | 15.1 (23.2, 33.8) |
| 2011-2012 (N=1657) | 13.2 (15.6, 18.2) | 6.8 (8.7, 11.2) | 52.8 (62.9, 71.9) | 10.0 (17.2, 28.0) |
| 2013-2014 (N=1769) | 15.6 (18.0, 20.6) | 8.7 (10.1, 11.8) | 47.8 (57.2, 66.1) | 6.8 (11.1, 17.5) |
| 2015-2016 (N=1691) | 14.9 (17.2, 19.7) | 8.1 (10.1, 12.5) | 44.0 (51.1, 58.2) | 10.8 (16.7, 25.0) |
| 2017-2020 (N=2973) | 15.5 (17.6, 20.0) | 8.1 (9.6, 11.4) | 48.8 (58.5, 67.6) | 12.8 (19.7, 29.0) |
| 2021-2023 (N=2368) | 17.6 (19.8, 22.2) | 10.8 (12.3, 14.0) | 39.8 (48.4, 57.0) | 7.8 (11.3, 16.0) |
| Average change per cycle^b^ | **0.66 (0.33, 0.99)** | **0.34 (0.08, 0.61)** | -0.58 (-2.44, 1.28) | -0.86 (-2.35, 0.63) |
| *P*-value for trend^b^ | **0.001** | **0.017** | 0.492 | 0.220 |
|  |  |  |  |  |
| **College graduate or above** |  |  |  |  |
| Total (N=15253) | 13.8 (13.1, 14.6) | 7.5 (6.9, 8.1) | 46.6 (42.3, 51.0) | 9.9 (7.9, 12.5) |
| 1999-2000 (N=793) | 9.6 (12.4, 15.9) | - | - | - |
| 2001-2002 (N=1078) | 8.7 (11.8, 15.7) | 5.2 (7.2, 9.7) | 30.9 (49.8, 68.7) | 12.0 (25.6, 46.5) |
| 2003-2004 (N=913) | 11.1 (12.9, 14.9) | 6.5 (8.1, 10.0) | 30.1 (49.8, 69.5) | 3.7 (14.7, 43.7) |
| 2005-2006 (N=978) | 10.2 (12.7, 15.8) | 6.0 (7.8, 10.0) | 47.2 (58.7, 69.3) | 7.3 (19.5, 42.7) |
| 2007-2008 (N=1098) | 10.4 (12.4, 14.6) | 4.4 (5.6, 7.2) | 34.9 (51.0, 66.9) | 4.0 (12.4, 32.7) |
| 2009-2010 (N=1258) | 9.2 (11.4, 14.0) | 4.1 (5.3, 6.9) | 30.9 (43.8, 57.7) | 1.4 (8.1, 35.8) |
| 2011-2012 (N=1396) | 12.5 (15.4, 18.9) | 5.8 (8.4, 12.2) | 38.3 (53.3, 67.7) | 4.6 (8.5, 15.1) |
| 2013-2014 (N=1441) | 12.3 (14.6, 17.1) | 6.7 (8.2, 10.0) | 29.2 (39.7, 51.2) | 7.0 (12.3, 20.9) |
| 2015-2016 (N=1421) | 12.6 (15.1, 18.0) | 6.5 (8.5, 11.0) | 33.7 (44.3, 55.4) | 1.2 (2.6, 5.6) |
| 2017-2020 (N=2256) | 11.8 (13.9, 16.4) | 5.1 (6.7, 8.6) | 26.1 (41.0, 57.9) | 3.9 (8.5, 17.6) |
| 2021-2023 (N=2621) | 15.2 (16.6, 18.2) | 7.8 (8.9, 10.2) | 37.5 (44.2, 51.0) | 4.9 (8.6, 14.8) |
| Average change per cycle^b^ | **0.39 (0.15, 0.63)** | 0.12 (-0.19, 0.44) | **-1.26 (-2.49, -0.03)** | **-1.70 (-2.80, -0.60)** |
| *P*-value for trend^b^ | **0.005** | 0.393 | **0.046** | **0.007** |

All prevalences and 95% CI were weighted.

^a^Due to inconsistencies in the question routing logic for three asthma-related items (current asthma, asthma attack in past year, and asthma-related emergency visits in past year) between the 1999-2000 cycle and other cycles, data from the 1999-2000 cycle were not used for these three asthma status variables.

^b^The estimate (β), 95% CI, and *P*-value for trend were obtained from linear regression models that included prevalence per cycle as the dependent variable and NHANES cycle as the continuous independent variable. The estimate (β) represents the average percentage change in prevalence per cycle.

Bold indicates significant estimates, showing statistically significant increasing or decreasing temporal trends in prevalence.

**eTable 9 Prevalence of different asthma statuses across cycles in the general population and PIR-stratified subgroups**

| Cycle | Lifetime asthma  (N=114505) | Current asthma^a^  (N=104744) | Current asthma | |
| --- | --- | --- | --- | --- |
|  |  |  | Asthma attack  in past year^a^  (N=9539) | Asthma-related emergency  visits in past year^a^  (N=7559) |
| **PIR <1.3** |  |  |  |  |
| Total (N=36910) | 17.3 (16.6, 17.9) | 11.2 (10.7, 11.8) | 52.0 (49.8, 54.3) | 24.2 (22.2, 26.4) |
| 1999-2000 (N=3154) | 11.7 (14.6, 18.0) | - | - | - |
| 2001-2002 (N=3405) | 13.2 (15.5, 18.1) | 8.4 (10.3, 12.7) | 47.6 (55.1, 62.3) | 26.1 (37.2, 49.9) |
| 2003-2004 (N=3360) | 13.1 (14.8, 16.7) | 7.9 (9.2, 10.7) | 50.0 (57.7, 65.1) | 25.1 (33.4, 42.8) |
| 2005-2006 (N=3143) | 13.2 (15.6, 18.4) | 8.6 (10.1, 12.0) | 45.8 (54.1, 62.2) | 25.5 (37.0, 50.2) |
| 2007-2008 (N=3199) | 14.7 (17.4, 20.6) | 9.2 (11.6, 14.7) | 53.0 (59.6, 65.8) | 23.9 (34.3, 46.4) |
| 2009-2010 (N=3559) | 14.7 (17.2, 20.0) | 9.1 (11.2, 13.8) | 43.6 (52.2, 60.7) | 31.1 (39.5, 48.6) |
| 2011-2012 (N=3506) | 15.0 (17.3, 19.7) | 9.6 (11.0, 12.6) | 45.5 (51.0, 56.4) | 13.0 (18.4, 25.4) |
| 2013-2014 (N=3676) | 16.8 (18.8, 21.0) | 10.1 (11.7, 13.4) | 40.5 (48.1, 55.9) | 14.9 (20.1, 26.5) |
| 2015-2016 (N=3056) | 17.2 (19.7, 22.5) | 10.5 (12.2, 14.1) | 48.3 (57.7, 66.6) | 17.1 (21.6, 27.0) |
| 2017-2020 (N=4252) | 16.9 (18.7, 20.6) | 10.4 (11.7, 13.2) | 36.2 (40.7, 45.3) | 14.2 (18.4, 23.6) |
| 2021-2023 (N=2600) | 17.6 (19.0, 20.6) | 11.3 (12.8, 14.6) | 44.3 (53.0, 61.4) | 13.6 (18.1, 23.6) |
| Average change per cycle^b^ | **0.51 (0.36, 0.66)** | **0.30 (0.16, 0.45)** | -0.95 (-2.22, 0.32) | **-2.53 (-3.87, -1.18)** |
| *P*-value for trend^b^ | **<0.001** | **0.001** | 0.124 | **0.002** |
|  |  |  |  |  |
| **PIR 1.3–3.5** |  |  |  |  |
| Total (N=37914) | 14.4 (13.9, 14.9) | 8.5 (8.1, 9.0) | 51.4 (48.8, 54.0) | 18.8 (16.5, 21.3) |
| 1999-2000 (N=2894) | 11.1 (13.5, 16.3) | - | - | - |
| 2001-2002 (N=3634) | 10.0 (11.7, 13.6) | 5.2 (6.6, 8.4) | 53.8 (61.7, 69.0) | 18.8 (27.0, 37.3) |
| 2003-2004 (N=3443) | 11.2 (12.7, 14.4) | 6.1 (7.4, 9.1) | 36.2 (45.5, 55.2) | 17.2 (27.8, 41.6) |
| 2005-2006 (N=3577) | 12.9 (14.2, 15.7) | 7.8 (8.5, 9.3) | 51.3 (59.9, 68.0) | 22.3 (34.2, 48.6) |
| 2007-2008 (N=3312) | 11.4 (12.8, 14.5) | 5.3 (6.7, 8.5) | 44.7 (53.3, 61.7) | 17.0 (25.8, 37.0) |
| 2009-2010 (N=3312) | 12.3 (13.9, 15.7) | 6.3 (7.3, 8.4) | 40.4 (45.5, 50.6) | 14.6 (25.1, 39.7) |
| 2011-2012 (N=2824) | 13.7 (16.4, 19.6) | 7.1 (9.4, 12.2) | 50.8 (60.6, 69.5) | 11.9 (19.5, 30.4) |
| 2013-2014 (N=2980) | 13.8 (15.4, 17.2) | 8.1 (9.7, 11.6) | 46.1 (54.2, 62.1) | 9.8 (16.3, 26.0) |
| 2015-2016 (N=3357) | 13.7 (15.1, 16.6) | 8.5 (9.5, 10.7) | 36.3 (43.8, 51.6) | 8.6 (12.4, 17.6) |
| 2017-2020 (N=4876) | 13.5 (14.9, 16.4) | 8.0 (9.0, 10.2) | 37.1 (45.5, 54.3) | 12.9 (17.8, 24.0) |
| 2021-2023 (N=3705) | 15.6 (17.4, 19.3) | 9.0 (10.5, 12.2) | 44.2 (50.2, 56.3) | 6.8 (9.7, 13.6) |
| Average change per cycle^b^ | **0.41 (0.19, 0.64)** | **0.38 (0.18, 0.58)** | -1.01 (-2.69, 0.67) | **-2.24 (-3.21, -1.27)** |
| *P*-value for trend^b^ | **0.003** | **0.003** | 0.205 | **0.001** |
|  |  |  |  |  |
| **PIR >3.5** |  |  |  |  |
| Total (N=27916) | 13.8 (13.1, 14.4) | 7.6 (7.1, 8.1) | 48.1 (45.4, 50.9) | 11.7 (9.9, 13.7) |
| 1999-2000 (N=2018) | 9.8 (11.9, 14.4) | - | - | - |
| 2001-2002 (N=2667) | 8.8 (11.0, 13.7) | 5.5 (6.7, 8.2) | 45.2 (56.7, 67.5) | 16.8 (23.2, 31.2) |
| 2003-2004 (N=2262) | 10.9 (12.2, 13.6) | 6.6 (7.7, 8.9) | 36.9 (47.1, 57.6) | 3.8 (10.4, 25.3) |
| 2005-2006 (N=2584) | 12.4 (14.4, 16.8) | 6.8 (8.3, 10.0) | 52.8 (61.2, 69.0) | 9.4 (16.1, 26.3) |
| 2007-2008 (N=2297) | 13.1 (14.6, 16.3) | 6.0 (7.3, 9.0) | 37.0 (45.6, 54.5) | 6.9 (13.3, 24.2) |
| 2009-2010 (N=2262) | 11.0 (12.3, 13.7) | 5.3 (6.1, 7.1) | 33.7 (43.0, 52.8) | 12.1 (20.6, 33.0) |
| 2011-2012 (N=2220) | 11.2 (14.1, 17.6) | 5.1 (7.6, 11.2) | 40.7 (49.7, 58.7) | 5.0 (8.5, 13.9) |
| 2013-2014 (N=2356) | 12.8 (14.5, 16.5) | 6.6 (7.8, 9.2) | 31.1 (41.2, 52.2) | 5.4 (9.4, 15.9) |
| 2015-2016 (N=2131) | 12.8 (14.4, 16.3) | 5.9 (7.6, 9.6) | 40.5 (48.6, 56.7) | 5.1 (10.7, 21.1) |
| 2017-2020 (N=3707) | 11.9 (14.1, 16.7) | 5.2 (6.9, 9.2) | 39.0 (46.7, 54.5) | 6.7 (10.0, 14.8) |
| 2021-2023 (N=3412) | 15.2 (16.7, 18.4) | 9.2 (10.2, 11.2) | 37.6 (42.6, 47.8) | 6.0 (9.7, 15.2) |
| Average change per cycle^b^ | **0.39 (0.16, 0.62)** | 0.15 (-0.11, 0.42) | -1.21 (-2.60, 0.18) | -1.06 (-2.14, 0.02) |
| *P*-value for trend^b^ | **0.004** | 0.219 | 0.080 | 0.053 |

All prevalences and 95% CI were weighted.

^a^Due to inconsistencies in the question routing logic for three asthma-related items (current asthma, asthma attack in past year, and asthma-related emergency visits in past year) between the 1999-2000 cycle and other cycles, data from the 1999-2000 cycle were not used for these three asthma status variables.

^b^The estimate (β), 95% CI, and *P*-value for trend were obtained from linear regression models that included prevalence per cycle as the dependent variable and NHANES cycle as the continuous independent variable. The estimate (β) represents the average percentage change in prevalence per cycle.

Bold indicates significant estimates, showing statistically significant increasing or decreasing temporal trends in prevalence.

**eTable 10 Prevalence of asthma medication use** **categorized by first level drug classification among asthmatic population across cycles**

| Cycle | Respiratory agents | Topical agents | Hormones/Hormone modifiers |
| --- | --- | --- | --- |
| Total (N=8465) | 42.5 (40.9, 44.2) | 8.9 (8.0, 9.8) | 1.6 (1.3, 2.0) |
| 1999-2000 (N=613) | 41.2 (36.6, 45.9) | 8.0 (4.9, 12.8) | 0.6 (0.2, 2.0) |
| 2001-2002 (N=698) | 41.9 (37.2, 46.7) | 6.8 (4.4, 10.4) | 2.5 (1.2, 5.3) |
| 2003-2004 (N=721) | 44.1 (40.7, 47.5) | 10.4 (7.8, 13.9) | 1.7 (1.0, 3.0) |
| 2005-2006 (N=775) | 45.6 (39.7, 51.6) | 12.5 (10.2, 15.3) | 2.6 (1.5, 4.5) |
| 2007-2008 (N=846) | 51.0 (46.0, 56.1) | 11.0 (8.2, 14.7) | 2.2 (1.5, 3.2) |
| Average change per cycle^a^  (1999-2008) | **2.34 (0.82, 3.86)** | 1.17 (-0.44, 2.79) | 0.34 (-0.43, 1.11) |
| *P*-value for trend^a^ | **0.016** | 0.104 | 0.256 |
| 2009-2010 (N=891) | 47.4 (42.4, 52.5) | 9.5 (6.8, 13.2) | 2.2 (1.4, 3.6) |
| 2011-2012 (N=860) | 44.6 (37.9, 51.6) | 8.3 (6.3, 10.9) | 0.9 (0.4, 1.8) |
| 2013-2014 (N=892) | 39.3 (34.7, 44.0) | 10.3 (7.0, 15.1) | 1.8 (1.2, 2.7) |
| 2015-2016 (N=836) | 40.5 (36.1, 45.0) | 7.9 (5.4, 11.3) | 1.0 (0.4, 2.8) |
| 2017-2020 (N=1333) | 35.2 (31.2, 39.3) | 5.2 (3.7, 7.3) | 1.1 (0.4, 3.2) |
| Average change per cycle^a^  (2009-2020) | **-2.87 (-4.60, -1.13)** | -0.91 (-2.47, 0.65) | -0.21 (-0.77, 0.35) |
| *P*-value for trend^a^ | **0.013** | 0.160 | 0.315 |
| Average change per cycle^a^ | -0.57 (-1.69, 0.54) | -0.20 (-0.76, 0.37) | -0.06 (-0.26, 0.13) |
| *P*-value for trend^a^ | 0.269 | 0.444 | 0.471 |

All prevalences and 95% CI were weighted. Drug classifications with overall prevalence greater than 1% were listed. Three-level therapeutic categories were obtained from the Multum Lexicon database.

^a^The estimate (β), 95% CI, and *P*-value for trend were obtained from linear regression models that included prevalence per cycle as the dependent variable and NHANES cycle as the continuous independent variable. The estimate (β) represents the average percentage change in prevalence per cycle.

Bold indicates significant estimates, showing statistically significant increasing or decreasing temporal trends in prevalence.

**eTable 11 Prevalence of asthma medication use categorized by second level drug classification among asthmatic population across cycles**

| Cycle | Bronchodilators | Nasal preparations | Leukotriene modifiers | Respiratory inhalant products | Adrenal cortical steroids |
| --- | --- | --- | --- | --- | --- |
| Total (N=8465) | 35.9 (34.2, 37.6) | 8.9 (8.0, 9.8) | 11.4 (10.4, 12.5) | 6.8 (6.1, 7.6) | 1.6 (1.3, 2.0) |
| 1999-2000 (N=613) | 33.4 (38.1, 43.1) | 4.9 (8.0, 12.8) | 5.0 (6.1, 7.5) | 8.0 (11.2, 15.5) | 0.2 (0.6, 2.0) |
| 2001-2002 (N=698) | 31.9 (37.3, 43.2) | 4.4 (6.8, 10.4) | 7.2 (9.1, 11.4) | 8.9 (11.8, 15.5) | 1.2 (2.5, 5.3) |
| 2003-2004 (N=721) | 34.9 (38.2, 41.5) | 7.8 (10.4, 13.9) | 9.0 (11.6, 14.8) | 4.7 (6.8, 9.7) | 1.0 (1.7, 3.0) |
| 2005-2006 (N=775) | 33.8 (39.1, 44.8) | 10.2 (12.5, 15.3) | 9.0 (12.6, 17.4) | 3.3 (5.6, 9.4) | 1.5 (2.6, 4.5) |
| 2007-2008 (N=846) | 39.7 (45.7, 51.9) | 8.2 (11.0, 14.7) | 9.5 (12.9, 17.2) | 4.4 (6.0, 8.2) | 1.5 (2.2, 3.2) |
| Average change per cycle^a^  (1999-2008) | 1.71 (-0.76, 4.18) | 1.17 (-0.44, 2.79) | **1.71 (0.63, 2.79)** | -1.66 (-3.32, 0.01) | 0.34 (-0.43, 1.11) |
| *P*-value for trend^a^ | 0.115 | 0.104 | **0.015** | 0.051 | 0.256 |
| 2009-2010 (N=891) | 38.7 (43.1, 47.6) | 6.8 (9.5, 13.2) | 9.1 (12.0, 15.6) | 4.6 (6.2, 8.3) | 1.4 (2.2, 3.6) |
| 2011-2012 (N=860) | 32.9 (39.3, 46.1) | 6.3 (8.3, 10.9) | 5.8 (8.9, 13.2) | 4.6 (6.9, 10.3) | 0.4 (0.9, 1.8) |
| 2013-2014 (N=892) | 26.9 (30.8, 35.0) | 7.0 (10.3, 15.1) | 8.1 (10.4, 13.4) | 4.6 (7.1, 10.8) | 1.2 (1.8, 2.7) |
| 2015-2016 (N=836) | 28.3 (32.8, 37.5) | 5.4 (7.9, 11.3) | 9.2 (12.6, 17.0) | 5.3 (7.2, 9.8) | 0.4 (1.0, 2.8) |
| 2017-2020 (N=1333) | 21.2 (25.8, 30.9) | 3.7 (5.2, 7.3) | 9.8 (12.6, 16.0) | 4.1 (5.5, 7.4) | 0.4 (1.1, 3.2) |
| Average change per cycle^a^  (2009-2020) | **-4.11 (-6.70, -1.52)** | -0.91 (-2.47, 0.65) | 0.50 (-1.14, 2.15) | -0.11 (-0.93, 0.71) | -0.21 (-0.77, 0.35) |
| *P*-value for trend^a^ | **0.015** | 0.160 | 0.403 | 0.706 | 0.315 |
| Average change per cycle^a^ | -1.10 (-2.39, 0.19) | -0.20 (-0.76, 0.37) | 0.40 (-0.11, 0.91) | **-0.47 (-0.93, -0.01)** | -0.06 (-0.26, 0.13) |
| *P*-value for trend^a^ | 0.085 | 0.444 | 0.109 | **0.048** | 0.471 |

All prevalences and 95% CI were weighted. Drug classifications with overall prevalence greater than 1% were listed. Three-level therapeutic categories were obtained from the Multum Lexicon database.

^a^The estimate (β), 95% CI, and *P*-value for trend were obtained from linear regression models that included prevalence per cycle as the dependent variable and NHANES cycle as the continuous independent variable. The estimate (β) represents the average percentage change in prevalence per cycle.

Bold indicates significant estimates, showing statistically significant increasing or decreasing temporal trends in prevalence.

**eTable 12 Prevalence of asthma medication use categorized by third level drug classification among asthmatic population across cycles**

| Cycle | Adrenergic  bronchodilators | Bronchodilator  combinations | Leukotriene modifiers^a^ | Nasal steroids | Inhaled  corticosteroids | Anticholinergic  bronchodilators | Glucocorticoids |
| --- | --- | --- | --- | --- | --- | --- | --- |
| Total (N=8465) | 29.1 (27.7, 30.5) | 12.6 (11.4, 13.8) | 11.4 (10.4, 12.5) | 8.4 (7.6, 9.3) | 6.5 (5.8, 7.3) | 2.7 (2.3, 3.3) | 1.6 (1.3, 2.0) |
| 1999-2000 (N=613) | 35.0 (30.8, 39.5) | 1.7 (0.9, 3.2) | 5.0 (6.1, 7.5) | 8.0 (4.9, 12.8) | 8.3 (6.2, 11.2) | 1.7 (1.1, 2.9) | 0.6 (0.2, 2.0) |
| 2001-2002 (N=698) | 34.0 (27.7, 40.8) | 4.2 (2.6, 6.5) | 7.2 (9.1, 11.4) | 6.7 (4.3, 10.3) | 10.1 (7.4, 13.5) | 0.7 (0.2, 3.0) | 2.5 (1.2, 5.3) |
| 2003-2004 (N=721) | 28.8 (26.2, 31.6) | 14.7 (11.3, 18.9) | 9.0 (11.6, 14.8) | 9.8 (7.2, 13.2) | 6.2 (4.3, 8.8) | 2.1 (1.2, 3.5) | 1.7 (1.0, 3.0) |
| 2005-2006 (N=775) | 32.7 (26.8, 39.2) | 14.6 (11.0, 19.1) | 9.0 (12.6, 17.4) | 12.0 (9.6, 14.8) | 5.4 (3.1, 9.2) | 2.5 (1.4, 4.3) | 2.6 (1.5, 4.5) |
| 2007-2008 (N=846) | 35.7 (30.4, 41.4) | 17.0 (13.8, 20.6) | 9.5 (12.9, 17.2) | 10.1 (7.4, 13.8) | 6.0 (4.4, 8.2) | 3.8 (2.9, 5.2) | 2.2 (1.5, 3.2) |
| Average change per cycle^b^  (1999-2008) | 0.01  (-3.16, 3.18) | **4.09**  **(1.13, 7.05)** | **1.71**  **(0.63, 2.79)** | 0.95  (-0.63, 2.53) | -0.93  (-2.43, 0.57) | 0.59  (-0.15, 1.34) | 0.34  (-0.43, 1.11) |
| *P*-value for trend^b^ | 0.993 | **0.022** | **0.015** | 0.151 | 0.144 | 0.086 | 0.256 |
| 2009-2010 (N=891) | 34.9 (30.7, 39.3) | 15.5 (12.1, 19.8) | 9.1 (12.0, 15.6) | 9.4 (6.7, 13.0) | 5.9 (4.3, 8.2) | 4.0 (2.3, 6.9) | 2.2 (1.4, 3.6) |
| 2011-2012 (N=860) | 31.8 (26.9, 37.3) | 13.5 (9.7, 18.6) | 5.8 (8.9, 13.2) | 7.6 (5.7, 10.2) | 6.9 (4.6, 10.3) | 2.4 (1.1, 5.1) | 0.9 (0.4, 1.8) |
| 2013-2014 (N=892) | 24.0 (20.5, 27.9) | 12.4 (10.3, 14.9) | 8.1 (10.4, 13.4) | 9.9 (6.5, 14.9) | 7.1 (4.6, 10.8) | 2.9 (1.9, 4.5) | 1.8 (1.2, 2.7) |
| 2015-2016 (N=836) | 26.9 (22.7, 31.5) | 12.1 (9.1, 15.8) | 9.2 (12.6, 17.0) | 7.9 (5.4, 11.3) | 7.2 (5.3, 9.7) | 2.9 (1.7, 4.8) | 1.0 (0.4, 2.8) |
| 2017-2020 (N=1333) | 20.8 (18.0, 23.9) | 10.3 (7.1, 14.8) | 9.8 (12.6, 16.0) | 4.6 (3.3, 6.4) | 5.5 (4.1, 7.4) | 2.6 (1.4, 4.7) | 1.1 (0.4, 3.2) |
| Average change per cycle^b^  (2009-2020) | **-3.32**  **(-5.95, -0.69)** | **-1.19**  **(-1.68, -0.71)** | 0.50  (-1.14, 2.15) | -0.92  (-2.60, 0.75) | -0.05  (-0.94, 0.83) | -0.23  (-0.82, 0.36) | -0.21  (-0.77, 0.35) |
| *P*-value for trend^b^ | **0.028** | **0.004** | 0.403 | 0.177 | 0.860 | 0.303 | 0.315 |
| Average change per cycle^b^ | **-1.24**  **(-2.19, -0.30)** | 0.71  (-0.50, 1.91) | 0.40  (-0.11, 0.91) | -0.21  (-0.74, 0.31) | -0.22  (-0.56, 0.13) | 0.17  (-0.06, 0.39) | -0.06  (-0.26, 0.13) |
| *P*-value for trend^b^ | **0.016** | 0.213 | 0.109 | 0.378 | 0.182 | 0.124 | 0.471 |

All prevalences and 95% CI were weighted. Drug classifications with overall prevalence greater than 1% were listed. Three-level therapeutic categories were obtained from the Multum Lexicon database.

^a^Leukotriene modifiers have no third-level classification, therefore the second level is presented.

^b^The estimate (β), 95% CI, and *P*-value for trend were obtained from linear regression models that included prevalence per cycle as the dependent variable and NHANES cycle as the continuous independent variable. The estimate (β) represents the average percentage change in prevalence per cycle.

Bold indicates significant estimates, showing statistically significant increasing or decreasing temporal trends in prevalence.

**eTable 13 Prevalence of asthma medication use categorized by pharmacological function among asthmatic population across cycles**

| Cycle | **SABA** | **ICS** | **LTRA** | **ICS+LABA** |
| --- | --- | --- | --- | --- |
| Total (N=8465) | 28.5 (27.1, 30.0) | 15.0 (13.9, 16.1) | 11.4 (10.4, 12.5) | 11.2 (10.2, 12.4) |
| 1999-2000 (N=613) | 33.3 (28.4, 38.5) | 15.3 (10.7, 21.6) | 6.1 (5.0, 7.5) | 0.0 (0.0, 0.0) |
| 2001-2002 (N=698) | 31.9 (26.0, 38.5) | 18.1 (14.3, 22.7) | 9.1 (7.2, 11.4) | 3.0 (1.6, 5.5) |
| 2003-2004 (N=721) | 27.1 (24.1, 30.3) | 16.4 (13.0, 20.4) | 11.6 (9.0, 14.8) | 12.9 (9.8, 16.9) |
| 2005-2006 (N=775) | 31.7 (25.7, 38.3) | 17.6 (14.3, 21.4) | 12.6 (9.0, 17.4) | 12.8 (9.7, 16.7) |
| 2007-2008 (N=846) | 35.5 (30.2, 41.1) | 15.6 (12.3, 19.7) | 12.9 (9.5, 17.2) | 13.9 (11.4, 16.8) |
| Average change per cycle^a^  (1999-2008) | 0.41 (-3.09, 3.90) | 0.01 (-1.40, 1.41) | **1.71 (0.63, 2.79)** | **3.76 (0.67, 6.85)** |
| *P*-value for trend^a^ | 0.736 | 0.991 | **0.015** | **0.031** |
| 2009-2010 (N=891) | 34.3 (30.2, 38.7) | 16.0 (13.4, 19.1) | 12.0 (9.1, 15.6) | 14.8 (11.3, 19.1) |
| 2011-2012 (N=860) | 31.8 (26.8, 37.2) | 14.4 (10.9, 18.8) | 8.9 (5.8, 13.2) | 12.3 (8.6, 17.3) |
| 2013-2014 (N=892) | 23.8 (20.3, 27.6) | 17.3 (14.4, 20.7) | 10.4 (8.1, 13.4) | 10.9 (8.2, 14.4) |
| 2015-2016 (N=836) | 26.7 (22.7, 31.0) | 14.6 (12.2, 17.2) | 12.6 (9.2, 17.0) | 11.2 (8.7, 14.3) |
| 2017-2020 (N=1333) | 20.8 (18.0, 23.9) | 10.3 (7.4, 14.0) | 12.6 (9.8, 16.0) | 9.7 (6.9, 13.5) |
| Average change per cycle^a^  (2009-2020) | **-3.22 (-5.89, -0.55)** | -1.14 (-3.41, 1.13) | 0.50 (-1.14, 2.15) | **-1.14 (-1.96, -0.31)** |
| *P*-value for trend^a^ | **0.031** | 0.209 | 0.403 | **0.022** |
| Average change per cycle^a^ | -1.01 (-2.02, 0.00) | -0.45 (-0.93, 0.02) | 0.40 (-0.11, 0.91) | 0.81 (-0.31, 1.94) |
| *P*-value for trend^a^ | 0.050 | 0.058 | 0.109 | 0.134 |
|  |  |  |  |  |
|  | **SABA+SAMA** | **LAMA** | **LABA** | **SAMA** |
| Total (N=8465) | 1.8 (1.4, 2.4) | 1.7 (1.3, 2.2) | 1.3 (1.0, 1.6) | 1.2 (0.9, 1.5) |
| 1999-2000 (N=613) | 1.7 (0.9, 3.2) | 0.0 (0.0, 0.0) | 4.8 (2.9, 7.9) | 1.7 (1.1, 2.9) |
| 2001-2002 (N=698) | 1.5 (0.8, 3.0) | 0.0 (0.0, 0.0) | 7.5 (4.7, 11.6) | 0.7 (0.2, 3.0) |
| 2003-2004 (N=721) | 3.0 (1.8, 5.1) | 0.1 (0.0, 1.2) | 2.8 (1.7, 4.6) | 1.9 (1.1, 3.3) |
| 2005-2006 (N=775) | 2.2 (0.9, 5.0) | 0.7 (0.2, 2.2) | 1.6 (0.9, 2.7) | 1.7 (0.9, 3.3) |
| 2007-2008 (N=846) | 4.0 (2.1, 7.4) | 2.4 (1.6, 3.6) | 0.8 (0.4, 1.5) | 1.9 (1.1, 3.4) |
| Average change per cycle^a^  (1999-2008) | 0.52 (-0.19, 1.23) | 0.54 (-0.06, 1.15) | -1.39 (-3.18, 0.40) | 0.14 (-0.39, 0.66) |
| *P*-value for trend^a^ | 0.101 | 0.064 | 0.090 | 0.474 |
| 2009-2010 (N=891) | 1.3 (0.7, 2.6) | 3.0 (1.5, 6.0) | 1.1 (0.4, 2.8) | 1.1 (0.4, 2.8) |
| 2011-2012 (N=860) | 1.9 (0.7, 4.8) | 1.6 (0.7, 3.7) | 0.6 (0.2, 2.0) | 0.8 (0.3, 1.9) |
| 2013-2014 (N=892) | 1.6 (0.6, 4.1) | 2.3 (1.4, 3.9) | 0.3 (0.0, 1.8) | 0.6 (0.2, 1.8) |
| 2015-2016 (N=836) | 1.7 (0.8, 3.8) | 2.1 (1.2, 3.6) | 0.6 (0.3, 1.0) | 0.9 (0.3, 2.6) |
| 2017-2020 (N=1333) | 0.5 (0.2, 1.2) | 2.0 (1.0, 4.0) | 0.0 (0.0, 0.4) | 1.0 (0.4, 2.0) |
| Average change per cycle^a^  (2009-2020) | -0.18 (-0.73, 0.37) | **-0.15 (-0.69, 0.38)** | -0.20 (-0.44, 0.03) | -0.01 (-0.23, 0.20) |
| *P*-value for trend^a^ | 0.367 | **0.431** | 0.071 | 0.868 |
| Average change per cycle^a^ | -0.12 (-0.36, 0.12) | **0.29 (0.09, 0.48)** | **-0.65 (-1.02, -0.27)** | -0.10 (-0.21, 0.02) |
| *P*-value for trend^a^ | 0.269 | **0.009** | **0.004** | 0.097 |

All prevalences and 95% CI were weighted. Drug pharmacological classifications with overall prevalence greater than 1% were listed.

^a^The estimate (β), 95% CI, and *P*-value for trend were obtained from linear regression models that included prevalence per cycle as the dependent variable and NHANES cycle as the continuous independent variable. The estimate (β) represents the average percentage change in prevalence per cycle. Bold indicates significant estimates, showing statistically significant increasing or decreasing temporal trends in prevalence.

Abbreviations: SABA, short-acting β2-agonists; LABA, long-acting β2-agonists; ICS, inhaled corticosteroids; SAMA, short-acting muscarinic antagonists; LAMA, long-acting muscarinic antagonists; LTRA, leukotriene receptor antagonists.

**eTable 14 Prevalence of top ten most commonly used asthma medications among asthmatic population across cycles**

| Cycle | **Albuterol** | **Montelukast** | **Fluticasone; Salmeterol** | **Fluticasone Nasal** | **Fluticasone** |
| --- | --- | --- | --- | --- | --- |
| Total (N=8465) | 27.2 (25.9, 28.6) | 11.1 (10.1, 12.3) | 8.5 (7.7, 9.4) | 5.2 (4.6, 6.0) | 4.1 (3.6, 4.6) |
| 1999-2000 (N=613) | 26.3 (31.1, 36.4) | 2.0 (3.2, 5.3) | 0.0 (0.0, 0.0) | 1.1 (2.7, 6.5) | 3.7 (5.8, 9.0) |
| 2001-2002 (N=698) | 25.4 (31.0, 37.3) | 6.1 (7.8, 9.8) | 1.6 (3.0, 5.5) | 1.3 (2.7, 5.4) | 6.5 (8.6, 11.4) |
| 2003-2004 (N=721) | 22.5 (25.2, 28.2) | 8.5 (11.1, 14.5) | 9.8 (12.9, 16.9) | 3.1 (4.9, 7.6) | 3.0 (4.4, 6.5) |
| 2005-2006 (N=775) | 23.4 (29.0, 35.2) | 9.0 (12.6, 17.4) | 9.7 (12.8, 16.7) | 3.7 (5.6, 8.2) | 1.8 (3.6, 7.1) |
| 2007-2008 (N=846) | 28.5 (33.8, 39.6) | 9.3 (12.8, 17.2) | 11.2 (13.6, 16.4) | 3.5 (4.7, 6.2) | 3.0 (4.1, 5.4) |
| Average change per cycle^a^  (1999-2008) | 0.34 (-3.32, 3.99) | **2.39 (0.73, 4.05)** | **3.71 (0.55, 6.87)** | 0.67 (-0.23, 1.56) | -0.85 (-2.61, 0.91) |
| *P*-value for trend^a^ | 0.788 | **0.020** | **0.033** | 0.098 | 0.223 |
| 2009-2010 (N=891) | 28.5 (32.3, 36.4) | 8.9 (11.8, 15.6) | 10.4 (13.0, 16.3) | 3.5 (5.9, 9.8) | 3.0 (4.3, 6.2) |
| 2011-2012 (N=860) | 25.7 (29.9, 34.4) | 5.8 (8.8, 13.2) | 6.1 (8.5, 11.8) | 4.5 (5.9, 7.6) | 2.7 (4.2, 6.5) |
| 2013-2014 (N=892) | 19.5 (22.9, 26.7) | 7.4 (10.1, 13.8) | 4.8 (6.8, 9.4) | 4.4 (6.9, 10.5) | 1.6 (2.8, 4.9) |
| 2015-2016 (N=836) | 22.2 (26.1, 30.4) | 9.2 (12.6, 17.0) | 3.6 (5.8, 9.1) | 4.3 (6.5, 9.8) | 2.3 (3.3, 4.8) |
| 2017-2020 (N=1333) | 17.9 (20.7, 23.8) | 9.8 (12.5, 15.9) | 2.8 (4.3, 6.5) | 2.9 (4.0, 5.6) | 2.5 (3.8, 5.7) |
| Average change per cycle^a^  (2009-2020) | **-2.69 (-5.21, -0.18)** | 0.51 (-1.15, 2.17) | **-2.03 (-3.25, -0.81)** | -0.31 (-1.44, 0.82) | -0.18 (-0.84, 0.47) |
| *P*-value for trend^a^ | **0.042** | 0.403 | **0.013** | 0.450 | 0.440 |
| Average change per cycle^a^ | -0.84 (-1.77, 0.09) | 0.61 (-0.05, 1.26) | 0.08 (-1.23, 1.39) | **0.31 (0.00, 0.61)** | **-0.37 (-0.69, -0.04)** |
| *P*-value for trend^a^ | 0.071 | 0.065 | 0.887 | **0.048** | **0.032** |
|  |  |  |  |  |  |
|  | **Budesonide; Formoterol** | **Mometasone Nasal** | **Beclomethasone** | **Albuterol; Ipratropium** | **Budesonide** |
| Total (N=8465) | 2.0 (1.6, 2.5) | 1.9 (1.5, 2.4) | 1.9 (1.5, 2.3) | 1.8 (1.4, 2.4) | 1.6 (1.3, 2.0) |
| 1999-2000 (N=613) | 0.0 (0.0, 0.0) | 1.9 (0.8, 4.6) | 2.5 (1.3, 4.8) | 1.7 (0.9, 3.2) | 0.6 (0.2, 2.0) |
| 2001-2002 (N=698) | 0.0 (0.0, 0.0) | 1.2 (0.6, 2.5) | 1.5 (0.6, 3.6) | 1.5 (0.8, 3.0) | 2.5 (1.2, 5.3) |
| 2003-2004 (N=721) | 0.0 (0.0, 0.0) | 2.9 (1.5, 5.4) | 1.0 (0.4, 2.5) | 3.0 (1.8, 5.1) | 1.7 (1.0, 3.0) |
| 2005-2006 (N=775) | 0.0 (0.0, 0.0) | 4.2 (2.5, 7.1) | 0.9 (0.2, 3.1) | 2.2 (0.9, 5.0) | 2.6 (1.5, 4.5) |
| 2007-2008 (N=846) | 0.3 (0.1, 1.0) | 2.6 (1.5, 4.5) | 1.8 (1.0, 3.0) | 4.0 (2.1, 7.4) | 2.2 (1.5, 3.2) |
| Average change per cycle^a^  (1999-2008) | 0.05 (-0.05, 0.16) | 0.45 (-0.56, 1.46) | -0.21 (-0.85, 0.44) | 0.52 (-0.19, 1.23) | 0.34 (-0.43, 1.11) |
| *P*-value for trend^a^ | 0.182 | 0.253 | 0.380 | 0.101 | 0.256 |
| 2009-2010 (N=891) | 1.9 (0.6, 5.8) | 2.1 (1.0, 4.6) | 1.2 (0.5, 2.9) | 1.3 (0.7, 2.6) | 2.2 (1.4, 3.6) |
| 2011-2012 (N=860) | 3.0 (1.6, 5.6) | 1.5 (0.6, 4.2) | 2.0 (1.1, 3.8) | 1.9 (0.7, 4.8) | 0.9 (0.4, 1.8) |
| 2013-2014 (N=892) | 3.0 (1.8, 5.0) | 2.1 (1.2, 3.7) | 3.0 (1.6, 5.6) | 1.6 (0.6, 4.1) | 1.8 (1.2, 2.7) |
| 2015-2016 (N=836) | 4.2 (3.3, 5.4) | 1.2 (0.6, 2.1) | 3.5 (2.2, 5.6) | 1.7 (0.8, 3.8) | 1.0 (0.4, 2.8) |
| 2017-2020 (N=1333) | 3.0 (1.9, 4.7) | 0.3 (0.1, 1.0) | 1.5 (1.0, 2.0) | 0.5 (0.2, 1.2) | 1.1 (0.4, 3.2) |
| Average change per cycle^a^  (2009-2020) | 0.34 (-0.37, 1.05) | -0.40 (-0.89, 0.08) | 0.20 (-0.88, 1.28) | -0.18 (-0.73, 0.37) | -0.21 (-0.77, 0.35) |
| *P*-value for trend^a^ | 0.227 | 0.078 | 0.602 | 0.367 | 0.315 |
| Average change per cycle^a^ | **0.50 (0.31, 0.68)** | -0.16 (-0.42, 0.10) | 0.10 (-0.11, 0.32) | -0.12 (-0.36, 0.12) | -0.06 (-0.26, 0.13) |
| *P*-value for trend^a^ | **<0.001** | 0.184 | 0.301 | 0.269 | 0.471 |

All prevalences and 95% CI were weighted. Drugs with overall prevalence greater than 1% were listed.

^a^The estimate (β), 95% CI, and *P*-value for trend were obtained from linear regression models that included prevalence per cycle as the dependent variable and NHANES cycle as the continuous independent variable. The estimate (β) represents the average percentage change in prevalence per cycle.

Bold indicates significant estimates, showing statistically significant increasing or decreasing temporal trends in prevalence.


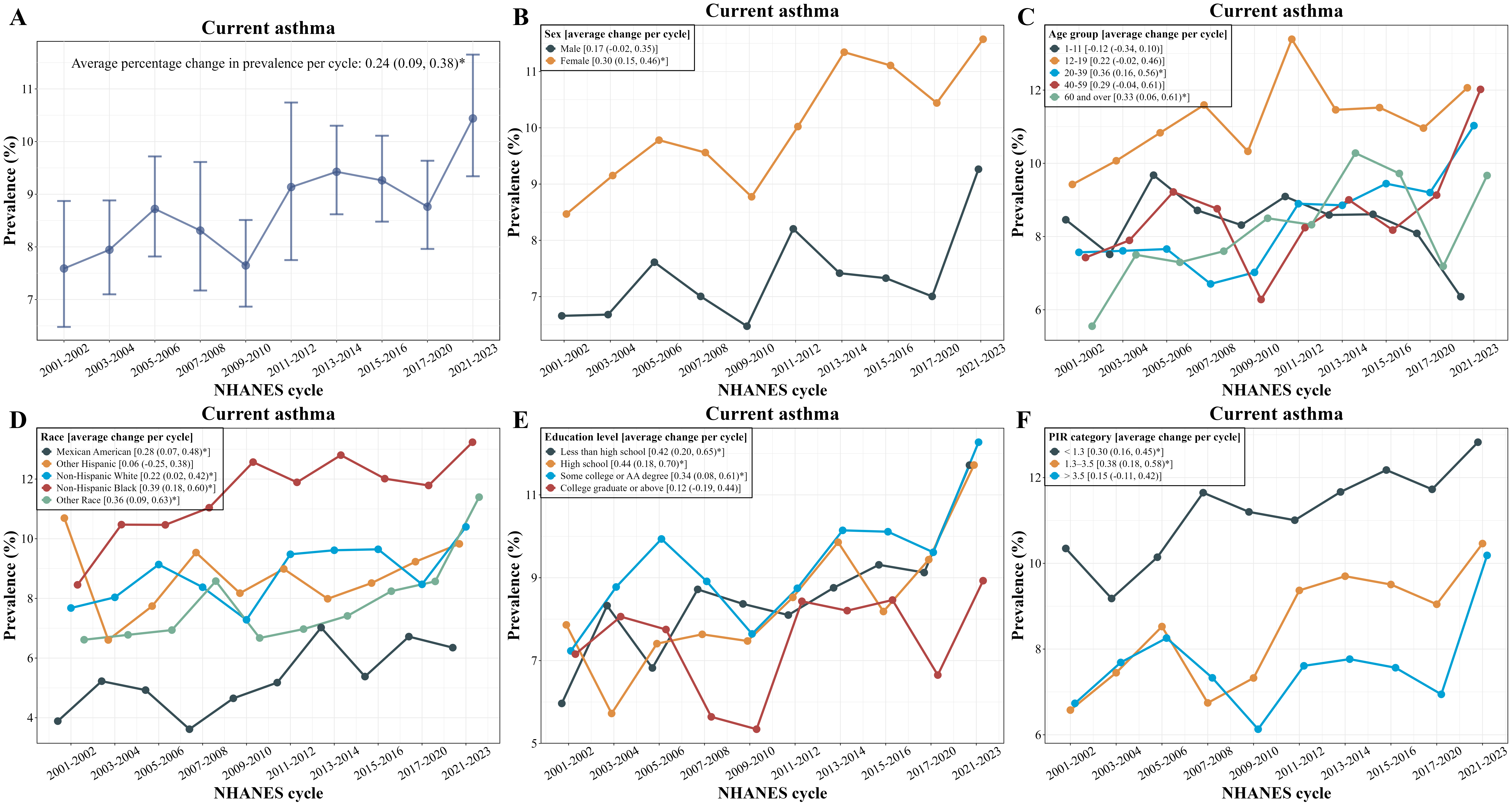


**eFigure 1 Trends in prevalence of current asthma in the general population and across demographic subgroups.** (A) Overall population; (B) Sex; (C) Age; (D) Race; (E) Educational attainment; (F) PIR.

The points on the line graph represent the weighted prevalence rate for each cycle. The error bars indicate the 95% CI for weighted prevalence. The estimate (β) and 95% CI in corresponding figure legends were obtained from linear regression models that included prevalence per cycle as the dependent variable and NHANES cycle as the continuous independent variable. The estimate (β) represents the average percentage change in prevalence per cycle. Asterisks indicate significant estimates, showing statistically significant increasing or decreasing temporal trends in prevalence.

Abbreviations: NHANES, National Health and Nutrition Examination Survey; PIR, poverty-to-income ratio.


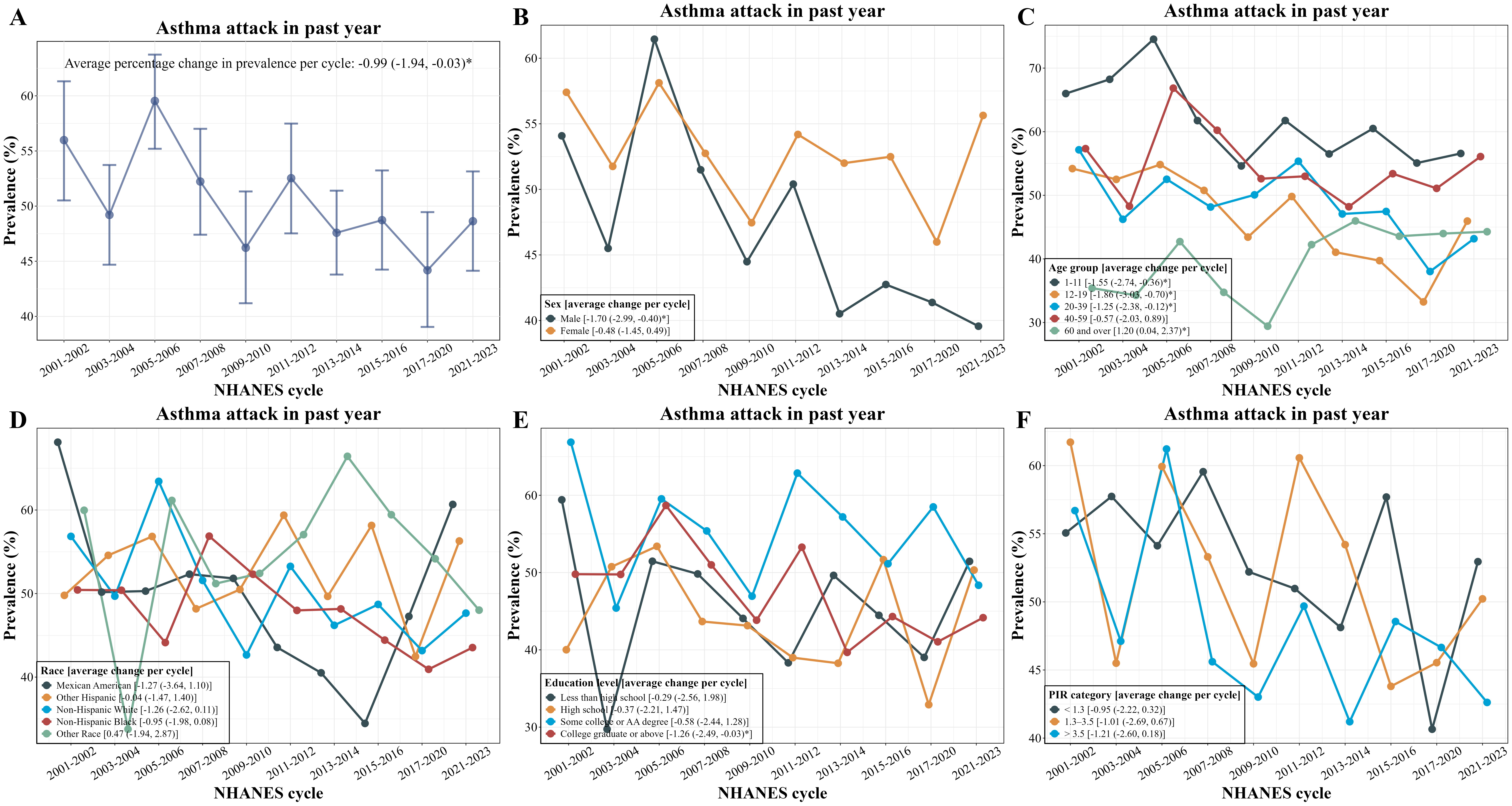


**eFigure 2 Trends in prevalence of asthma attack in past year in the general population and across demographic subgroups.** (A) Overall population; (B) Sex; (C) Age; (D) Race; (E) Educational attainment; (F) PIR.

The points on the line graph represent the weighted prevalence rate for each cycle. The error bars indicate the 95% CI for weighted prevalence. The estimate (β) and 95% CI in corresponding figure legends were obtained from linear regression models that included prevalence per cycle as the dependent variable and NHANES cycle as the continuous independent variable. The estimate (β) represents the average percentage change in prevalence per cycle. Asterisks indicate significant estimates, showing statistically significant increasing or decreasing temporal trends in prevalence. The error bars indicate the 95% CI for weighted prevalence.

Abbreviations: NHANES, National Health and Nutrition Examination Survey; PIR, poverty-to-income ratio.


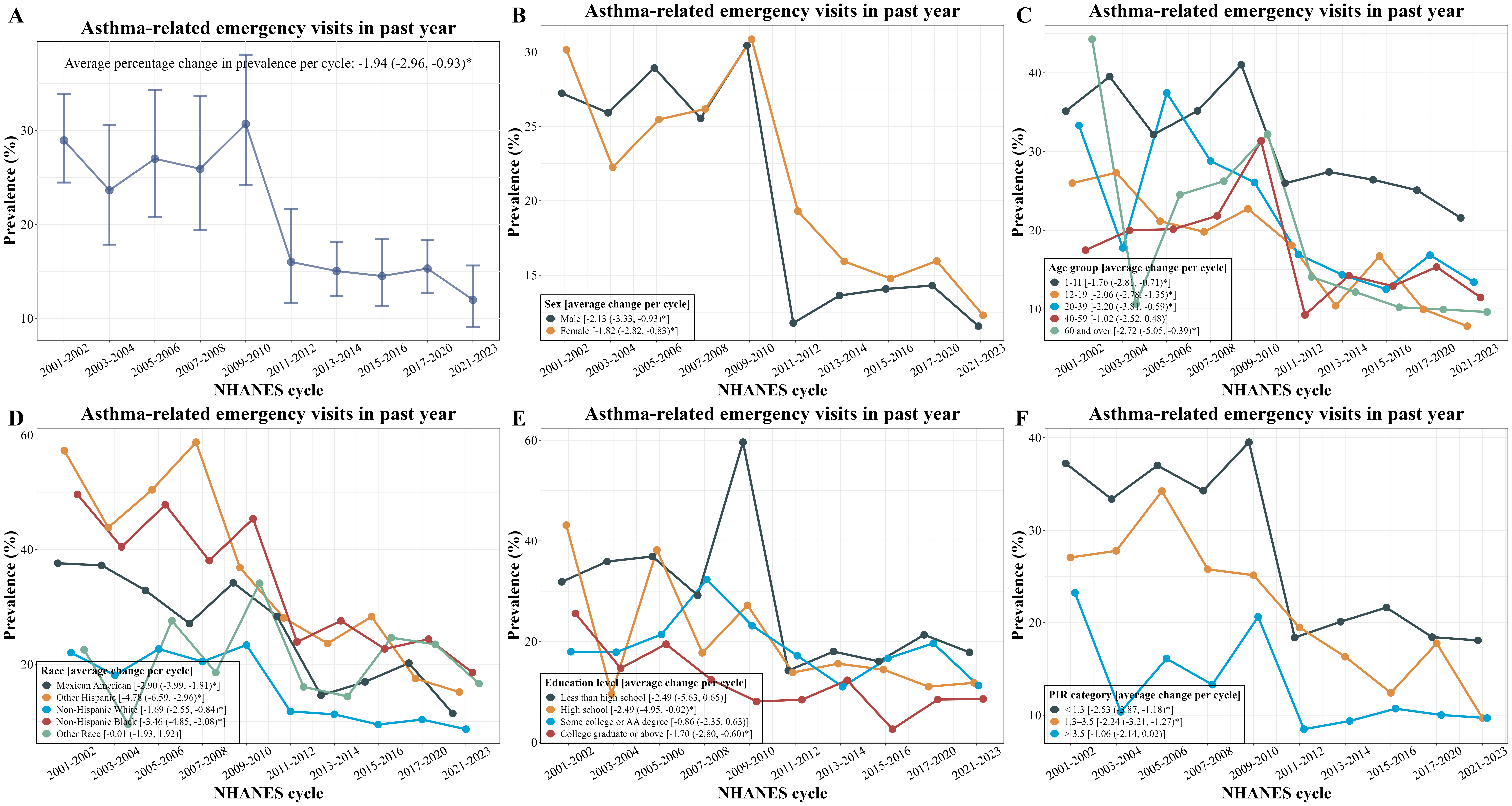


**eFigure 3 Trends in prevalence of asthma-related emergency visits in past year in the general population and across demographic subgroups.** (A) Overall population; (B) Sex; (C) Age; (D) Race; (E) Educational attainment; (F) PIR.

The points on the line graph represent the weighted prevalence rate for each cycle. The error bars indicate the 95% CI for weighted prevalence. The estimate (β) and 95% CI in corresponding figure legends were obtained from linear regression models that included prevalence per cycle as the dependent variable and NHANES cycle as the continuous independent variable. The estimate (β) represents the average percentage change in prevalence per cycle. Asterisks indicate significant estimates, showing statistically significant increasing or decreasing temporal trends in prevalence. The error bars indicate the 95% CI for weighted prevalence.

Abbreviations: NHANES, National Health and Nutrition Examination Survey; PIR, poverty-to-income ratio.


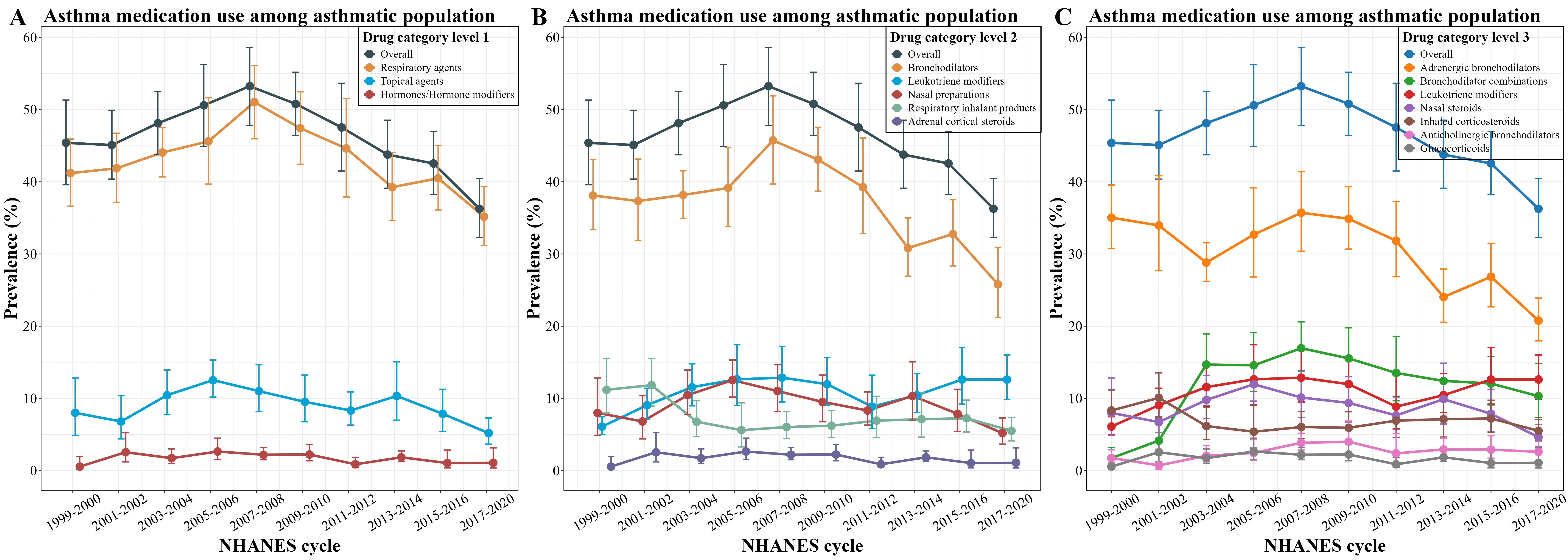


**eFigure 4 Trends in prevalence of asthma medication use categorized by second-level drug classification.** (A) First level; (B) Second level; (C) Third level. Three-level therapeutic categories were obtained from the Multum Lexicon database.

The points on the line graph represent the weighted prevalence rate for each cycle. The error bars indicate the 95% CI for weighted prevalence. At the bottom of each panel: The estimate (β) and 95% CI in corresponding figure legends were obtained from linear regression models that included prevalence per cycle as the dependent variable and NHANES cycle as the continuous independent variable. The estimate (β) represents the average percentage change in prevalence per cycle. Asterisks indicate significant estimates, showing statistically significant increasing or decreasing temporal trends in prevalence. Due to distinct shifts in asthma medication trends around 2007-2008, separate calculations were performed for 1999-2008 and 2009-2020. Bold indicates significant estimates, showing statistically significant increasing or decreasing temporal trends in prevalence.

Abbreviations: NHANES, National Health and Nutrition Examination Survey; PIR, poverty-to-income ratio.
